# eHealth in TB clinical management

**DOI:** 10.5588/ijtld.21.0602

**Published:** 2022-12-01

**Authors:** I. Margineanu, C. Louka, O. Akkerman, Y. Stienstra, J-W. Alffenaar

**Affiliations:** 1 Department of Clinical Pharmacy and Pharmacology, University Medical Centrum Groningen, University of Groningen, Groningen, the Netherlands; 2 Iasi Pulmonary Diseases University Hospital, Iasi, Romania; 3 Department of Internal Medicine/Infectious Diseases, University Medical Center Groningen, University of Groningen, Groningen, the Netherlands; 4 Tuberculosis Center Beatrixoord, University Medical Center Groningen, University of Groningen, Groningen, the Netherlands; 5 Department of Pulmonary Diseases and Tuberculosis, University Medical Center Groningen, University of Groningen, Groningen, the Netherlands; 6 Department of Clinical Sciences, Liverpool School of Tropical Medicine, Liverpool, UK; 7 Faculty of Medicine and Health, School of Pharmacy, University of Sydney, Camperdown, NSW, Australia; 8 Westmead Hospital, Sydney, NSW, Australia; 9 Marie Bashir Institute for Infectious Diseases and Biosecurity, University of Sydney, Sydney, NSW, Australia

**Keywords:** tuberculosis, telemedicine, respiratory, clinical

## Abstract

**BACKGROUND:**

The constant expansion of internet and mobile technologies has created new opportunities in the field of eHealth, or the digital delivery of healthcare services. This TB meta-analysis aims to examine eHealth and its impact on TB clinical management in order to formulate recommendations for further development.

**METHODS:**

A systematic search was performed using the Preferred Reporting Items for Systematic Reviews and Meta-Analyses framework in PubMed and Embase of articles published up to April 2021. Screening, extraction and quality assessment were performed by two independent researchers. Studies evaluating an internet and/or mobile-based eHealth intervention with an impact on TB clinical management were included. Outcomes were organised following the five domains described in the WHO “Recommendations on Digital Interventions for Health System Strengthening” guideline.

**RESULTS:**

Search strategy yielded 3,873 studies, and 89 full texts were finally included. eHealth tended to enhance screening, diagnosis and treatment indicators, while being cost-effective and acceptable to users. The main challenges concern hardware malfunction and software misuse.

**CONCLUSION:**

This study offers a broad overview of the innovative field of eHealth applications in TB. Different studies implementing eHealth solutions consistently reported on benefits, but also on specific challenges. eHealth is a promising field of research and could enhance clinical management of TB.

## Abstract (fr)

**CONTEXTE:**

L’expansion constante d’Internet et des technologies mobiles a créé de nouvelles opportunités en matière d’e-Santé ou de fourniture numérique de services de santé. Cette méta-analyse sur la TB s’est penchée sur les solutions d’e-Santé et leur impact sur la prise en charge clinique de la TB afin de formuler des recommandations en faveur de leur développement.

**MÉTHODES:**

Une analyse systématique des articles publiés jusqu’en avril 2021 a été réalisée sur PubMed et Embase, en utilisant la liste PRISMA (*Preferred Reporting Items for Systematic Reviews and Meta-Analyses*). L’examen, la sélection et l’évaluation de qualité des articles ont été réalisés par deux investigateurs indépendants. Les études ayant évalué une intervention d’e-Santé sur Internet et/ou sur mobile avec un impact sur la prise en charge clinique de la TB ont été incluses. Les résultats ont été organisés selon les cinq domaines décrits dans les recommandations de l’OMS intitulées « Recommendations on Digital Interventions for Health System Strengthening ».

**RÉSULTATS:**

Nous avons identifié 3 873 études, et 89 textes entiers ont finalement été inclus. Les solutions de e-Santé avaient tendance à renforcer les indicateurs relatifs au dépistage, au diagnostic et au traitement, tout en étant rentables et acceptables pour les utilisateurs. Les problèmes principaux étaient dysfonctionnement du matériel informatique et mauvaise utilisation des logiciels.

**CONCLUSION:**

Cette étude fournit un vaste aperçu du domaine innovant que représentent les applications de e-Santé pour la TB. Plusieurs études ayant mis en place des solutions de e-Santé en ont rapporté les avantages, mais aussi les problèmes spécifiques. L’e-Santé est un domaine de recherche prometteur et pourrait permettre de renforcer la prise en charge clinique de la TB.

In the past 25 years, the growing availability of internet-based technologies has alteered the global landscape.^1^ In 2021, 60% of the world’s population has internet access, 2 billion of whom are in low-income countries, steadily closing the gap in internet and cellular access.^2^ The medical world has begun to take advantage of these technologies by employing increasingly more internet and mobile solutions which expand, assist or enhance medical activities, a field known as digital health or electronic health (eHealth).^3^ The WHO has recently published the “Global Strategy on Digital Health 2020–2025” report, highlighting the requirements for successful implementation of digital health, and encouraging the development of this field in a sustainable, equitable and transparent manner.^4^

Emerging technologies are especially attractive for TB, as they could provide cost-efficient, practical, innovative solutions^5^–^7^ for an infectious disease that primarily affects low and lower-income countries.^8^ However, as with every relatively new research field, interventions have been experimental, employing different technologies, and study designs, and covering multiple aspects of TB management.

The field of eHealth in TB is gaining pace, as recognised by the End TB strategy,^9^ which expressly mentions the “application of novel information and communication technologies for health”, in support of future eHealth developments. Furthermore, the recent guideline “Recommendations on Digital Interventions for Health System Strengthening”^10^ addresses some of these issues regarding heterogeneity by offering a framework to organise, inform and guide stakeholders and policy-makers about the role of eHealth interventions in healthcare delivery.

Efforts to organise the field of eHealth in TB and to provide recommendations for future development are under way; however, recent reviews have either been narrative,^11^ or focused on certain aspects of TB care.^12^ This systematic review addresses multiple calls to organise this new field, including from the European Commission, and especially in the present global context,^13^,^14^ and aims to offer a “birds-eye” view on implemented eHealth interventions in TB care to understand their application, opportunities and challenges in order to provide recommendations for future development of eHealth in TB care.

## METHODS

A research protocol was developed according to the Preferred Reporting Items for Systematic Reviews and Meta-Analyses (PRISMA) framework and registered in the international prospective register of systematic reviews (PROSPERO registration number CRD42018115440). A systematic literature search was performed of the PubMed and EMBASE databases, with a final check in April 2021.

eHealth interventions were defined as any solution which employed internet and/or mobile devices, deployed in a clinical medical setting. Two authors (IM, CL) independently screened all papers’ titles and abstracts. Unresolved conflicts were resolved by a third reviewer (JWC). Covidence software (Covidence; Melbourne VIC, Australia) was used for screening and quality evaluation, MS Excel 2013 (Microsoft, Redmond, WA, USA) for data extraction and Rev Man 5.4 (Cochrane, London, UK) for meta-analysis.

### Search queries

Known synonyms and word clouds for “eHealth” were used in the search query. Full search query can be found in the Supplementary Data.

### Study selection

Inclusion criteria were usage of internet and/or mobile technologies, implementation analysis, user base with at least one of TB current or former patients, TB contacts, medical staff involved in TB clinical care (nurses, physicians, para-medical staff), with comparisons. Studies were included regardless of language used, basic demographics or type of study. Grey literature, any type of reviews, policy papers, books were excluded. For studies without full text access, the original authors were contacted.

### Outcome measures

Outcome measures were grouped following the WHO “Recommendations on digital interventions for health system strengthening” evidence-to-decision framework,^10^ which generated the five main domains under which all outcomes were nested. Effectiveness included diagnosis and treatment indicators, such as adherence and cure rates. Acceptability referred to outcomes pertaining to user perceptions of the intervention, such as acceptability and user satisfaction with the intervention. Feasibility covered challenges and facilitators for the interventions. Resource use pertained to cost-effectiveness. Gender, rights, equality focused on privacy and patient support.

### Data extraction and quality assessment

Data extraction included first author, year of publication, country, type of study, type of intervention, PICO (population, intervention, comparison, outcome) criteria, and GRADE criteria for quality assessment.^15^ Two authors (IM, CL) independently performed data extraction and quality assessment, where outcomes were graded by taking into consideration the overall quality of the studies included, based on the GRADE quality of evidence criteria.^16^ Publication bias was analysed using funnel plots, and on an individual study basis by evaluating the publications themselves.

### Data synthesis and analysis

Meta-analysis was performed on studies with similar populations and outcome measures, and different analyses were performed depending on outcome: studies reporting dichotomous data were analysed using the random-effects odds ratio (OR) Mantel-Haenzel method, with their 95% confidence intervals (CIs); this model was chosen based on the assumption that there might be other factors influencing the outcome beyond the intervention itself. Studies reporting continuous data were analysed using inverse-variance random effects, and expressed in mean difference, 95% CIs. Studies reporting diagnosis accuracy were included in a diagnosis accuracy review and described as a forest plot (including specificity and sensitivity of each diagnosis method included) and a summary receiver operating characteristics (SROC) plot. All outcomes not in meta-analysis were reported as a narrative synthesis. An Excel file was compiled using all study data based on outcomes. All costs were harmonised in 2021 euros using an inflation calculator^17^ and the current exchange rate.

## RESULTS

### General results

**Figure 1:**
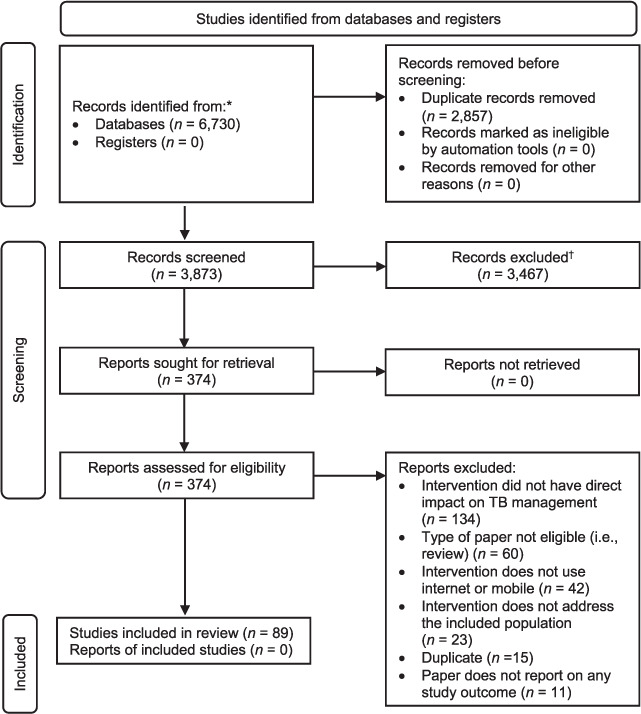
PRISMA 2020 flow diagram for new systematic reviews (included searches of databases and registers only). *Consider reporting the number of records identified from each database or register searched if possible (rather than the total number across all databases/registers). ^†^If automation tools were used, indicate how many records were excluded by a human and how many were excluded using automation tools. Source: Page MJ, et al. The PRISMA 2020 statement: an updated guideline for reporting systematic reviews. BMJ 2021;372:n71. PRISMA = Preferred Reporting Items for Systematic Reviews and Meta-Analyses.

Search queries resulted in 3,873 studies eligible for screening, of which 89 were included in our review (Figure 1). Only six full texts (1.5%) warranted the additional opinion of a third reviewer. One study was not written in English,^18^ and was translated using the authors’ research network. Of the 89 studies, 17 were randomised controlled trials (RCTs), 7 cluster RCTs, 21 non-RCTs, and 44 used a “before and after” design. By country of implementation, the largest proportion of studies were from the United States (*n* = 12), followed by South Africa (*n* = 11).

**Figure 2:**
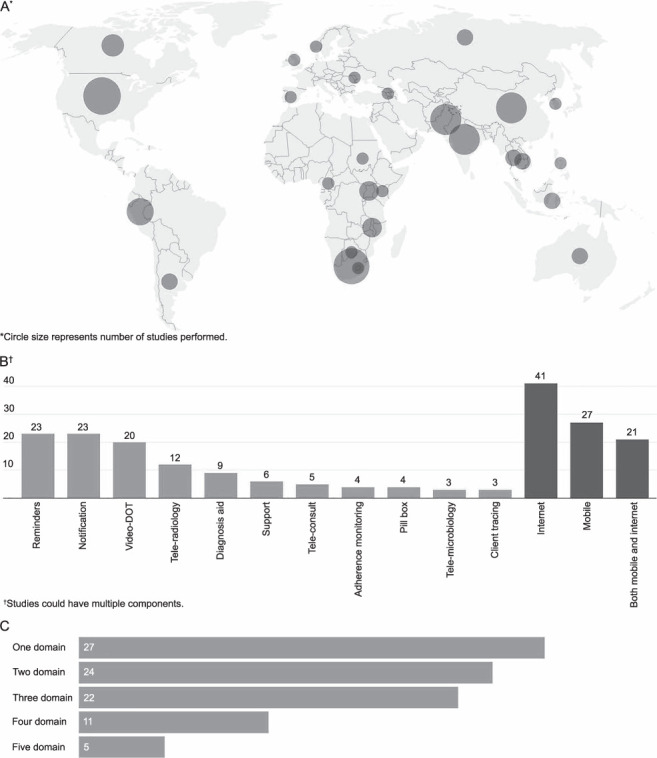
Study characteristics: A) countries where eHealth studies were performed;* B) studies by intervention components;^†^C) studies analysed by WHO domain.

There has been an increase in the number of studies performed as the years progressed, with a maximum of four studies published per year before 2015 to 18 studies in 2020; the quality of the studies improved at the same time: before 2015, there were 7 (22%) cluster RCTs, but 17/57 (30%) after 2015. Using the GRADE criteria for quality of evidence, 35 (39%) had very low, 32 (36%) low, 7 (8%) moderate and 15 (17%) high. There was no clear publication bias identified as funnel plots indicate differences in results and there were negative results across studies. Of the 89 studies, 86 (93.3%) reported no conflict of interest and 3 studies reported authors setting up small academic companies to collect royalties from their proposed interventions.^19^–^21^ The majority of studies analysed a maximum of three WHO domains, with only five reporting outcomes on all five domains (Figure 2).

### Effectiveness domain

eHealth tended to enhance diagnosis procedures at each step of the diagnosis cascade. Through eHealth, TB diagnoses could be made where there was a lack of expertise, with one study reporting that a remote panel of experts had helped avoid “22 wrong treatment schemes”.^22^ Second, our meta-analysis indicated that eHealth increased the likelihood of a person to be correctly referred, resulting in a higher chance of initiating treatment in a timely manner (Table), with one study observing that the intervention increased the number of microbiological samples correctly referred 275-fold (from 9 to 2,479).^23^

**Table:** Summary of outcomes

Quality of evidence	Outcome summary
Domain: effectiveness	
Referrals: moderate	8 studies;^43^–^50^ total events in intervention: 5,405/919,090; total events in control: 2,517/1,217,270. meta-analysis, random effects, favours intervention: OR for a person to be correctly referred in intervention vs. standard of care: 4.38 (95% CI 2.35–8.19), τ^2^ 0.79, *I*^2^ = 97%
Diagnosis performance: moderate	5 studies;^19^,^51^–^54^ total number of patients: 17,925; meta-analysis, diagnosis accuracy, sROC analysis, radiology automated diagnosis tools tend to outperform standard of care (physician) (Figure 3). Diagnosis of TB established through sputum culture or GeneXpert (Cepheid, Sunnyvale, CA, USA)
Diagnosis narrative: outcomes	Tele-radiology,^55^–^59^ tele-microscopy^60^,^61^ with human experts aiding the diagnosis: concordance of 74.7–100% with standard of care. Automated radiology^24^,^62^ and TST readers^29^ accuracy depend on cut-off points, ranging from 20% to 95%. Automated TB diagnosis aids have sensitivity of 66.7% and 89%^30^,^63^
Patients adherence: moderate	27 studies;^5^,^21^,^32^,^33^,^35^,^38^,^45^,^64^–^83^ total events in intervention: 3,400/5,414; total events in control: 4,177/6,824; meta-analysis, OR, random effects, favours intervention: OR for a person to complete TB treatment in intervention vs. standard of care: 1.79 (95% CI 1.33–2.40), τ^2^ 0.35, *I*^2^ = 85%, *Z* = 3.89
Adherence: FEDO: moderate	8 studies;^5^,^18^,^21^,^37^,^67^,^83^–^85^ total patients in intervention: 681; total patients in control: 704; meta-analysis, mean difference, favours intervention: patients in intervention groups had a higher observable fraction with 10.9% than standard of care (95% CI 0.75–20.97), τ^2^ 200, *I*^2^ = 99%, *Z* = 2.11
Adherence: FEDO: narrative outcomes	FEDO reported as the same between groups by one study,^86^ better in intervention groups in 7 studies,^5^,^37^,^76^,^87^,^88^ and worse in intervention by one study (underutilised app because of the reported errors)^34^
Cure rate: moderate	8 studies;^33^,^34^,^38^,^73^,^74^,^79^,^81^,^89^ total events in intervention: 1,249/2,259; total events in control: 1,248/2,633; meta-analysis, OR, random effects, favours intervention: OR for patients in intervention groups to be cured 1.45 (95% CI 1.08–1.94) vs. standard of care τ^2^ 0.09, *I*^2^ = 68%, *Z* = 2.49
Cure rate narrative	Cure rate was lower in intervention (11% vs. 30%, the app was misused by patients and health providers alike)^35^
Sputum conversion narrative	Patients in intervention groups had faster sputum conversion by 16 days in one study,^25^ more patients had sputum conversion at 2 months in three studies.^45^,^78^,^90^ One study reported that less non-MDR patients in intervention sputum converted at 2 months^90^
Error rate: low	There were between 10% and 97% less errors in intervention vs. standard of care (paper forms)^22^,^38^,^91^,^92^
Intervention additional benefits narrative	13 studies;^22^,^30^,^37^,^66^,^67^,^70^,^76^,^85^–^87^,^93^–^95^ in order of frequency: flexibility (4), improved communication (4), convenience (3), the possibility of individualising the intervention (2), less medical staff exposed to active cases (1), improvement of the knowledge base (1)
Domain: acceptability	
User satisfaction: low	15 studies;^7^,^18^,^21^,^26^,^37^,^49^,^57^,^71^,^81^,^83^,^84^,^94^,^96^–^98^ between 61% and 100% of the participants would recommend the intervention 90.3%;^25^,^85^,^89^ users in intervention groups scored higher on satisfaction scores, 3 studies: 99.5% vs. 99.2%, 100% vs. 70%; 92% vs. 88% 3 studies;^70^,^86^,^87^ narrative: “Satisfaction in intervention groups 3.29 higher than control”,^87^ “high satisfaction in intervention group”,^57^,^72^,^86^ “overall, satisfaction was higher in intervention than in control”^70^
Intervention perceived usefulness: low	10 studies;^20^,^21^,^32^,^71^,^73^,^75^,^81^,^82^,^95^–^97^ between 79% and 100% found intervention useful Medical staff agreed intervention was useful, 1 study;^99^ more users in intervention groups found the intervention useful, 2 studies: 96% vs. 56.6%, 80% vs 32%^71^,^81^ 2 studies;^32^,^92^ usefulness scores: 7.5/10 and 7.7/10
Domain: feasibility	
Hardware challenges narrative	Most frequent hardware challenges: broken equipment or dead batteries, 7 studies:^68^,^72^,^75^,^76^,^88^,^94^,^97^ shared phones, 4 studies: stolen phone, 2 studies: 75,100
Software challenges: low	Software-related incidents, 9 studies: up to 10% (0.7–8%) of missed videos or messages;^18^,^20^,^26^,^33^,^59^,^68^,^76^,^97^,^101^,^102^ software challenges: messages not sent, 2 studies^46^,^103^ consults not being performed, 1 study (initially 25%, dropped to 8% after learning curve^47^ “system freeze”, “software quirks”, “server down”^18^,^20^,^26^,^33^,^59^,^68^,^76^,^97^,^101^,^102^
Network/electricity challenges: very low	Network-related issues, 17 studies: interruptions between 2 days and 8 weeks, “lower adherence correlated with poor network coverage”, “slow internet”^20^,^30^,^32^,^37^,^50^,^59^,^72^,^75^,^76^,^85^,^86^,^88^,^92^,^94^,^97^,^103^,^104^ 4 studies;^66^,^75^,^88^,^97^ reports on electricity outages causing issues “for several participants”
User base-specific challenges narrative: very low	Lack of comprehension/training about the intervention, 10 studies^20^,^34^,^66^,^68^,^75^,^76^,^88^,^93^,^97^ not knowing phone number, 4 studies^47^,^50^,^75^,^100^ preference for face-to-face contact, 5 studies^33^,^35^,^46^,^60^,^80^,^86^ scheduling conflicts and forgetfulness, 4 studies,^18^,^76^,^85^,^94^ other user challenges, 3 studies: “more interest in computers than in the intervention”, “no trickle-down effect”^21^,^22^,^91^
Domain: resource use	
Cost-saving: low	Medical facilities saved costs, 3 studies: between €13.5 and €13,495.7 per patient in travel and personnel costs;^5^,^7^,^20^,^62^,^65^,^84^,^85^,^102^,^105^,^106^ patients saved costs, 4 studies: between €1.5 and €75 in travel costs;^71^,^81^,^87^,^106^ costs saving, other: the break-even point would be 2.9–5.5 years,^101^ “if one is willing to pay $2, the probability of cost-effectiveness rises to almost 90%”,^37^ costs per session associated with live vDOT (€6.54), recorded-vDOT (€5.35), clinic DOT (€8.46) and field-DOT (€19.83)^106^

**Table:** (continued)

Quality of evidence	Outcome summary
Mileage-saving: low	Saved 2,368 km and 454.93 km per patient. 2 studies,^85^,^103^ interventions are especially useful where travel would be a necessity; 7 studies^7^,^21^,^22^,^32^,^39^,^76^,^94^
Capacity-saving: low	6 studies;^39^,^43^,^44^,^50^,^76^,^102^ interventions allowed medical facilities to increase their capacity (“see more patients”): between 100% and 208%
Time-saving: moderate	5 studies;^5^,^18^,^81^,^92^,^106^ total patients in intervention: 2,042; total patients in control: 3,139; meta-analysis, mean difference, favours intervention: intervention consults and observed doses were faster with a mean difference of 11.25 min (95% CI 8.57–13.92) than standard of care, τ^2^ 14.28, *I*^2^ = 99%, *Z* = 8.24
Time-saving narrative	Saved time, 4 studies, 2.93–3.1 min saved per sample,^101^ between 19.7 min and 3.24 h saved per consult^22^,^23^,^25^,^26^,^38^,^39^,^44^,^57^,^59^,^70^,^71^,^86^,^87^,^102^,^107^,^108^ less visits per patient were required in intervention: from 38,160 to 4,604 (decrease of 87.9%).^99^1 study: intervention was 7x slower (small field of view in tele-microscopy)^60^
Domain: gender, equality, rights	
Education: very low	Increase knowledge scores, 2 studies: of 12%, 21%;^75^,^96^ No difference in knowledge scores: 3 studies^30^,^64^,^73^
Patient support narrative	Patients felt “cared for by staff”,^32^,^80^ “80.9% family supporters reported that phone calls helped them feel confident that the disease was under control”^89^, 1 study mentions no difference in support levels between intervention and control^82^
Privacy: low	3 studies;^26^,^37^,^71^ 2–27% of users worried about privacy breaches 56.6–100% users felt the intervention was better at protecting their privacy than control^7^,^18^,^20^,^21^,^30^,^49^,^70^,^71^,^84^–^86^,^101^ 8 studies;^21^,^26^,^32^,^49^,^66^,^75^,^90^,^95^ there were zero privacy breaches for 819 participants (vs. one privacy breach in one study in the control group)

OR = odds ratio; CI = confidence interval; sROC = summary receiver operating characteristic; TST = tuberculin skin test; FEDO = fraction of observed dose; vDOT = video directly observed therapy.

**Figure 3:**
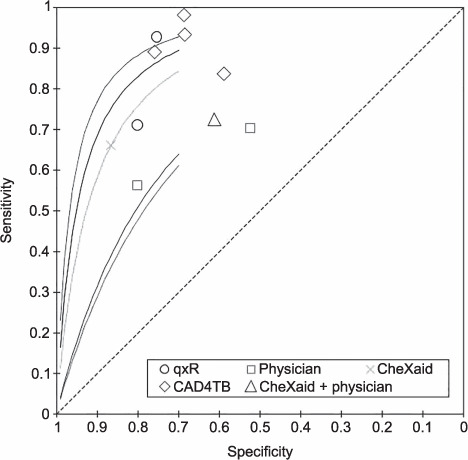
sROC plot of automated X-ray diagnosis aids. sROC = summary receiver operating characteristic.

In recent years, the most robust analyses concerning diagnosis have focused on artificial intelligence/machine learning programmes dedicated to radiology, and meta-analysis suggests that these supersede standard of care (Figure 3, Supplementary Figure S1). Two studies not only compared automated diagnosis aids to genomic tests, but also to physicians, and concluded that the interventions superseded standard of care. However, studies mention several caveats, such as the fact that automated TB scoring would depend on cut-off points, and that diagnosis accuracy might be lower for certain diagnoses such as “hilar adenopathy” and “consolidation”.^24^

With regard to treatment indicators (Table, Supplementary Figures S2 and S3), digital health tended to perform better—meta-analysis results indicated that the OR of a patient completing treatment within the eHealth group was higher than standard of care and that patients in the eHealth group had a higher observable fraction overall. Furthermore, meta-analysis reports on a higher cure rate for the eHealth group. Two studies analysing the same app, 99DOTS, reported generally worse outcomes, explained by the misuse of the app by both patients and medical providers alike, which was attributed to a lack of training and dissemination.

eHealth introduction reduced error rates in medical charts and in laboratory results compared to standard of care, such as incorrect bacteriological results or medication doses. Various studies also noted additional benefits of eHealth interventions such as “less paperwork”, “automatic response to frequent questions”, “viewing all patient information on one page”, “fewer nurses exposed to TB” and “increased reporting of side effects”.

### Acceptability domain

**Figure 4:**
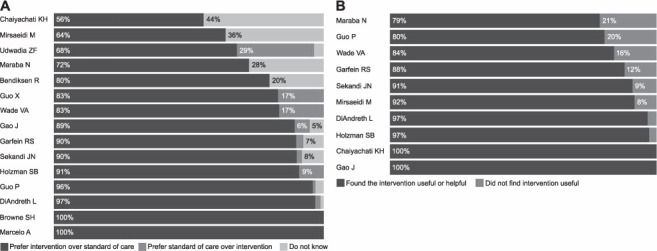
Users who prefer interventions and who find interventions useful: A) percentage of participants who prefer interventions over standard of care; B) percentage of participants who found interventions useful.

Overall, users were satisfied with the interventions, measured either qualitatively or quantitatively. Satisfaction scores were higher in intervention groups and most participants would prefer or would recommend the interventions (Figure 4). Most users perceived the interventions as useful.

### Feasibility domain

Four main types of challenges were reported by studies: hardware, software, network/electricity and user base. Hardware availability was reported as an issue in a minority of cases, with most users experiencing malfunction or battery drain. Software issues were easier to resolve, with one study mentioning that there were “1.13 technical issues a month, which the medical staff could fix themselves”,^25^ and another that “use improved with experience”.^26^ Network interruptions and limited internet bandwidth caused several studies to report issues with data transmission; however, out of 17 studies reporting on these issues, 15 were before 2020. The most important user-related challenge is not understanding how the intervention works, with one study mentioning that “problems were resolved in 77.6–91.8% of cases” through training. In one study with automated SMS reminders, 28% of users did not always understand the message due to technical language.

### Resource use domain

**Figure 5:**
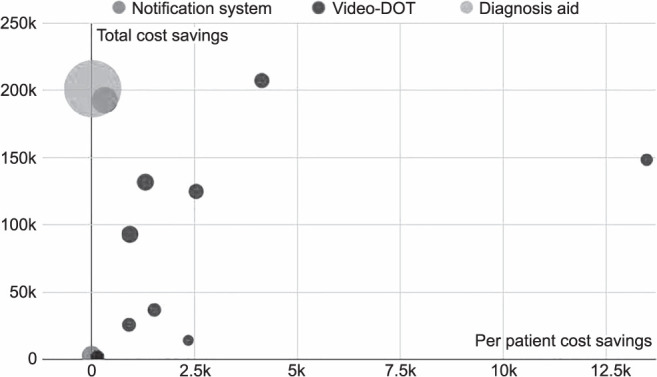
Cost-savings of medical facilities. vDOT = video directly observed therapy.

None of the included studies concluded that eHealth interventions are more costly than standard of care, with all reporting various degrees of savings, depending on the local economy and travel time, translated in work hours saved, and resources it would take to reach the patients (Figure 5).

Interventions also saved time, the most notable differences being in communicating between different medical specialties, particularly when results were sent and consultations performed via postal services. Furthermore, as indicated also by our results, interventions reduced travel and consultation time in case of directly observed therapy, which, in turn, led some facilities to attend to more patients per unit of time.

Studies mentioned that patients felt “cared for by staff”: “80.9% family supporters reported that phone calls helped them feel confident that the disease was under control”. Intervention users generally believed that the intervention offered greater privacy than standard of care; there were no reports of breaches of confidentiality in the studies.

## DISCUSSION

This systematic review evaluated eHealth applicability for TB prevention and treatment using the framework of the “Recommendations on digital interventions for health system strengthening” and aggregated data from 89 clinical trials. With the passage of time, research in the subject expanded, the field got more established, and studies became more rigorous. This phenomenon was observed by another systematic review.^12^

Overall, interventions tended to be non-inferior or perform slightly better regarding diagnosis and treatment indicators. An earlier systematic review investigated the role of mobile phones in HIV-TB management, which demonstrated a positive effect on medication adherence,^27^ while another systematic review found no difference in adherence.^28^ Unsurprisingly, effectiveness of interventions depends on the setting and level of care in which they are tested.

Results suggest that diagnosis using tele-medicine are feasible to implement in current practice, especially in locations lacking TB expertise. Other diagnosis apps, such as the automated tuberculin skin test reader, can be used only with the aid of skilled medical personnel.^29^ To note, medical chart error rate dropped in all studies which quantified it, and additional benefits, such as reduced workloads, were mentioned as early as 1999: “electronic information resources eliminated bulky manuals and charts. Nurses also reported greater empowerment”.^30^

Benefits felt by users were reflected in the acceptability domain, where the majority of users found interventions useful and more satisfactory than standard of care. This could be especially relevant in the current context of the COVID-19 pandemic, which has reduced potential and current patient’s healthcare access, leading to a decrease in TB detection and a loss of adherence.^31^ One study specifically included extensively drug-resistant patients in danger of losing medication and consultations during the COVID-19 crisis in India and concluded that “while inexpensive and expedient, telemedicine may risk compromising the quality of care associated with a physical examination; however, in times of COVID-19, this is a trade-off we may have to accept.” However, if we analyse healthcare worker and patient user preference for face-to-face contact under the “acceptability” domain, we note that there is a minority of studies with users citing this preference.

Implementing eHealth is not without its challenges; this has also been mentioned by an analysis of the landscape and research priorities in eHealth.^11^ Overall, impactful hardware issues happened primarily in low and middle-income countries, besides dead phone batteries which happened everywhere. Stolen, broken, shared, or not having a phone were noted in six low-income countries (LICs) and 11 middle-income countries (MICs). The same distribution was observed for network failures, with only 6/17 studies being conducted in high-income countries. Electricity outages all happened in LICs or MICs.

User-related challenges were reported by most studies and were the most diverse, ranging from users not knowing their own phone number^32^,^33^ to “the requesting physician appeared to take more interest in computers than in the medical diagnosis”.^22^ Regarding the user base, multiple studies noted that successful implementation was dependent on the user’s tech savviness and, barring that, their education. The best example of this cautionary tale is the 99DOTS app, with multiple studies reporting that its misuse and underuse because of a lack of training led to inefficiencies in its implementation.^34^,^35^

The domain where eHealth shined was cost-effectiveness. Introduction of medication monitors and video-observed therapy were expected to lead to substantial cost savings.^36^ This forecast was supported by the results presented in the resource use domain, with considerable savings per patient, especially in travel time and costs for either the patient or medical staff, but also because internet consultations tended to be more efficient, with one nurse noting “it was easier to finish videophone visits, as the patients did not try to prolong calls by offering a cup of tea or social interaction”.^37^ Furthermore, interventions also led to streamlined sample transportation and result communication so much so that one study reported that “because of the delay, patients as well as his or her physicians often forgot that they had ever performed a culture” before eHealth implementation.^38^ Finally, eHealth tended to make better use of human resources, with one study specifically mentioning that the capacity of medical facilities increased, “without a reduction in the volume of [control] encounters”.^39^

A minority of patients mentioned being worried about privacy breaches, but the majority consider digital health to be safer than controls. One study noted that while 58% of medical staff worried about unintentional disclosure of private files, 87% of patients were not worried at all about confidentiality breaches. There were no reported breaches of privacy in the studies included (vs. one in a control group). It appears that if professionals approach eHealth in TB care with the same rigour they approach any other professional medical data, users can trust them to keep their data confidential.

Last but not least, as TB is a stigmatising, lonely disease,^40^ it is important to highlight the studies which reported that patients “no longer felt isolated”,^41^ “were happy when receiving motivational texts”,^42^ and felt “cared for by staff”.^32^ However, no studies reported on gender, sexual or race inequalities.

Overall, it is important to note that while eHealth was at least non-inferior concerning effectiveness, users trusted and were satisfied with the interventions; eHealth implementation was also cost-effective if potential challenges are taken into account. eHealth interventions could be especially relevant in the current context.

### Strengths

This systematic review organises a broad body of evidence and offers an overview of the five WHO domains for analysing eHealth. The focus of this review was clinical care, and by analysing the interplay between the five domains, it can offer guidance on the challenges to be resolved before implementing eHealth and provide information on potential benefits, especially those pertaining to user perception, patient data safety and cost-effectiveness.

### Limitations

As eHealth is a relatively new field, earlier studies tended to have a historical cohort or the same cohort as a comparison group. Also, methods to analyse eHealth impact have evolved, from simple interviews to standardised questionnaires and economic analysis. However, only five studies analysed all WHO domains. Grey literature was not accessed, as a cursory search revealed that it tended to skim on outcome reporting. Quality of evidence varied, with the best evidence in the effectiveness domain, and the least in the gender, equality and rights domain.

## CONCLUSION

eHealth adoption in TB is growing and most eHealth interventions fulfil the five WHO domains goals. Interventions tended to add value to standard of care, measured by “hard” indicators of effectiveness and resource use, but also by “soft” indicators of acceptability. eHealth interventions are especially useful where travel is required and in settings with a lack of resources and expertise. However, infrastructure, experience and training are needed to ensure that eHealth is effective. Nevertheless, as the global trend is towards the increasing use of technology in everyday life, users will become savvier, health interventions will become more readily available, and evidence more robust and reliable.
