# Supplementary material for: eHealth in TB clinical management
Source: Int J Tuberc Lung Dis. 2022 Dec 1;26(12):1151–61. doi: 10.5588/ijtld.21.0602 (PMC9728950; doi:10.5588/ijtld.21.0602)
Supplement: Supplementary file 1 [file iutld_ijtld_21.0602_supplementarydata1.pdf]

http://dx.doi.org/10.5588/ijtld.21.0602

eHealth in TB clinical management

SUPPLEMENTARY DATA

Search strings

Pubmed search

("Telemedicine"[Mesh] OR "Distance Counseling"[Mesh] OR "Internet"[Mesh] OR "Mobile Applications"[Mesh] OR "Cell Phone"[Mesh] OR "Electronic Mail"[Mesh] OR e-health[tiab] OR ehealth[tiab] OR m-health[tiab] OR mhealth[tiab] OR telemedicine[tiab] OR app[tiab] OR apps[tiab] OR mobile[tiab] OR phone*[tiab] OR smartphone[tiab] OR tablet[tiab] OR tablets[tiab] OR web-based[tiab] OR webbased[tiab] OR online[tiab] OR internet[tiab] OR e-therap*[tiab] OR e-program*[tiab] OR software[tiab] OR sms[tiab] OR text messaging[tiab] OR email[tiab] OR e-mail[tiab])

AND

("Tuberculosis"[Mesh] OR tuberculosis[tiab])

Embase search

('telemedicine'/de OR 'teleconsultation'/exp OR 'telediagnosis'/exp OR 'telemonitoring'/exp OR 'telepathology'/exp OR 'teleradiology'/exp OR 'telerehabilitation'/exp OR 'teletherapy'/exp OR 'e-counseling'/exp OR 'Internet'/exp OR 'mobile application'/exp OR 'mobile phone'/exp OR (‘e-health’ OR ehealth OR ‘m-health’ OR mhealth OR telemedicine OR app OR apps OR mobile OR phone* OR smartphone OR tablet OR tablets OR ‘web-based’ OR webbased OR online OR internet OR ‘e-therap*’ OR ‘e-program*’ OR software OR sms OR ‘text messaging’ OR email OR ‘e-mail’):ab,ti)

AND

('tuberculosis'/exp OR tuberculosis:ab,ti)

NOT

('conference abstract'/it OR 'conference paper'/it OR 'editorial'/it OR 'letter'/it OR 'note'/it)

Supplementary Figure S1: Meta-analysis on diagnosis accuracy, forest plots

TP - true positive; FP - false positive; FN - false negative; TN - true negative

qXR

Study	TP	FP	FN	TN	Sensitivity (95% CI)	Specificity (95% CI)
Khan 6057	252	475	20	1451	0.93 [0.89, 0.95]	0.75 [0.73, 0.77]
Nash 5802	225	122	92	490	0.71 [0.66, 0.76]	0.80 [0.77, 0.83]

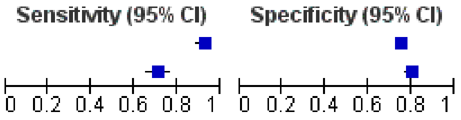

CAD4TB

Study	TP	FP	FN	TN	Sensitivity (95% CI)	Specificity (95% CI)
Habib 5880	62	254	12	366	0.84 [0.73, 0.91]	0.59 [0.55, 0.63]
Khan 6057	254	603	18	1323	0.93 [0.90, 0.96]	0.69 [0.67, 0.71]
Murphy 5857	854	1487	104	4711	0.89 [0.87, 0.91]	0.76 [0.75, 0.77]
Philipsen 5649	293	3257	5	7194	0.98 [0.96, 0.99]	0.69 [0.68, 0.70]

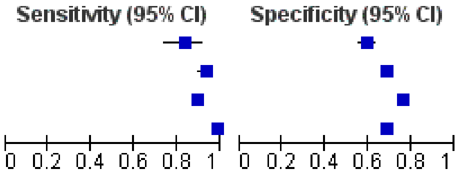

physician

Study	TP	FP	FN	TN	Sensitivity (95% CI)	Specificity (95% CI)
Nash 5802	178	122	139	490	0.56 [0.50, 0.62]	0.80 [0.77, 0.83]
Rajpurkar 6092	33	32	14	35	0.70 [0.55, 0.83]	0.52 [0.40, 0.65]

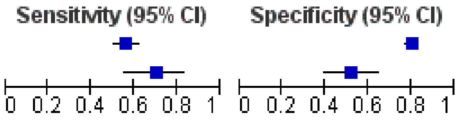

CheXaid + physician

Study	TP	FP	FN	TN	Sensitivity (95% CI)	Specificity (95% CI)
Rajpurkar 6092	34	26	13	41	0.72 [0.57, 0.84]	0.61 [0.49, 0.73]

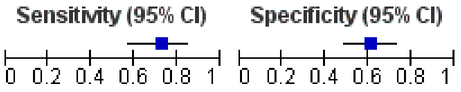

CheXaid

Study	TP	FP	FN	TN	Sensitivity (95% CI)	Specificity (95% CI)
Rajpurkar 6092	31	9	16	58	0.66 [0.51, 0.79]	0.87 [0.76, 0.94]

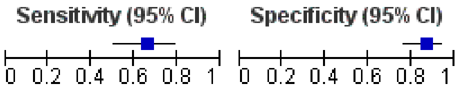

Comparison of different imaging artificial intelligence algorithms. TB diagnosis confirmation obtained with sputum cultures (Khan 6057, Nash 5802), Xpert MTB/RIF (Habib 5880, Murphy 5857, Philipsen 5649), or both (Rajpurkar 6092). Meta-analysis used was diagnosis test accuracy, which analyses different diagnosis tests true and false positives and true and false negatives in order to obtain an overall sensitivity and specificity. Because of the inherent

heterogeneity of such studies, a random effects model is usually chosen. Forest plots graphically illustrate the data presented, giving an estimation of the distance from the value of 1, and a visual representation of the confidence intervals the different studies reported.

Supplementary Figure S2: Meta-analysis, forest plots

Fig. S2-A: Forest plot of "Referrals" outcome

Study or Subgroup	Intervention Events	Intervention Total	Control Events	Control Total	Weight	Odds Ratio M-H, Random, 95% CI
Chadha, 2017	127	194	67	194	12.1%	3.59 [2.36, 5.46]
Choun, 2017	108	109	1213	1948	5.6%	65.44 [9.12, 469.80]
Davis, 2019	7	471	5	448	8.9%	1.34 [0.42, 4.24]
DiAndreth L	127	174	19	226	11.5%	29.44 [16.54, 52.41]
Khan, 2012	3140	915767	496	1213226	12.8%	8.41 [7.65, 9.25]
Long, 2015 (1)	497	623	126	200	12.3%	2.32 [1.64, 3.28]
Long, 2015 (2)	503	669	241	362	12.5%	1.52 [1.15, 2.01]
Wagstaff , 2019	331	405	78	101	11.7%	1.32 [0.78, 2.24]
Wang, 2009	565	678	272	565	12.5%	5.39 [4.15, 6.99]
**Total (95% CI)**		**919090**		**1217270**	**100.0%**	**4.38 [2.35, 8.19]**
Total events	5405		2517			

Heterogeneity: $Tau^2 = 0.79$; $Chi^2 = 251.94$, df = 8 ($P < 0.00001$); $I^2 = 97\%$
Test for overall effect: $Z = 4.63$ ($P < 0.00001$)

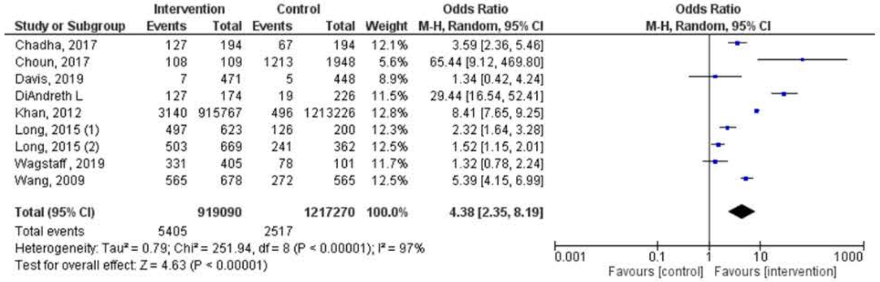

Fig. S2-B: Forest plot of "Treatment adherence" outcome

Study or Subgroup	Intervention Events	Intervention Total	Control Events	Control Total	Weight	Odds Ratio M-H, Random, 95% CI
Ali, 2019	69	74	69	74	2.9%	1.00 [0.28, 3.61]
Bassett, 2016	75	194	89	212	5.7%	0.87 [0.59, 1.30]
Belknap, 2017	240	315	237	321	5.8%	1.13 [0.79, 1.62]
Browne, 2019	32	41	0	20	0.9%	140.26 [7.74, 2541.64]
Chen 2020	53	80	98	160	5.2%	1.24 [0.71, 2.18]
Chuck, 2016	47	49	260	267	2.2%	0.63 [0.13, 3.14]
Das Gupta, 2020	184	222	104	111	4.2%	0.33 [0.14, 0.76]
Fang, 2017	154	160	165	190	3.9%	3.89 [1.55, 9.74]
Faroqui, 2017	49	74	49	74	4.7%	1.00 [0.51, 1.98]
Guo P, 2020	202	203	198	202	1.4%	4.08 [0.45, 36.83]
Guo X, 2020	148	235	7	158	4.3%	36.70 [16.44, 81.89]
Hermans, 2017	128	183	224	302	5.7%	0.81 [0.54, 1.22]
Hirsch-Moverman, 2017	163	183	132	166	5.0%	2.10 [1.15, 3.82]
Holzman, 2018	10	28	4	28	2.8%	3.33 [0.90, 12.36]
Holzschuh, 2017	15	15	12	12		Not estimable
Iribarren, 2013	17	18	17	19	1.1%	2.00 [0.17, 24.19]
Johnston, 2018	117	153	148	183	5.3%	0.77 [0.45, 1.30]
Khachadourian, 2929	187	187	172	198	0.9%	57.61 [3.48, 952.55]
Kumboyono, 2017	42	45	36	45	2.6%	3.50 [0.88, 13.92]
Lam, 2018	44	50	196	302	4.0%	3.97 [1.64, 9.61]
Lee, 2011	62	91	210	366	5.4%	1.59 [0.98, 2.59]
Long, 2015 (1)	150	150	656	691	0.9%	16.28 [0.99, 266.83]
Mohammed 2016	332	1104	325	1093	6.2%	1.02 [0.85, 1.22]
Ratchakit-Nedsuwan, 2020	39	40	37	40	1.3%	3.16 [0.31, 31.78]
Story, 2019	78	122	35	114	5.2%	4.00 [2.32, 6.89]
Thekkur P, 2019	510	870	443	961	6.2%	1.66 [1.38, 1.99]
Yoeli, 2019	285	569	254	535	6.1%	1.11 [0.88, 1.41]
**Total (95% CI)**		**5455**		**6844**	**100.0%**	**1.79 [1.33, 2.40]**
Total events	3432		4177			

Heterogeneity: $Tau^2 = 0.35$; $Chi^2 = 168.47$, df = 25 ($P < 0.00001$); $I^2 = 85\%$
Test for overall effect: $Z = 3.89$ ($P < 0.0001$)

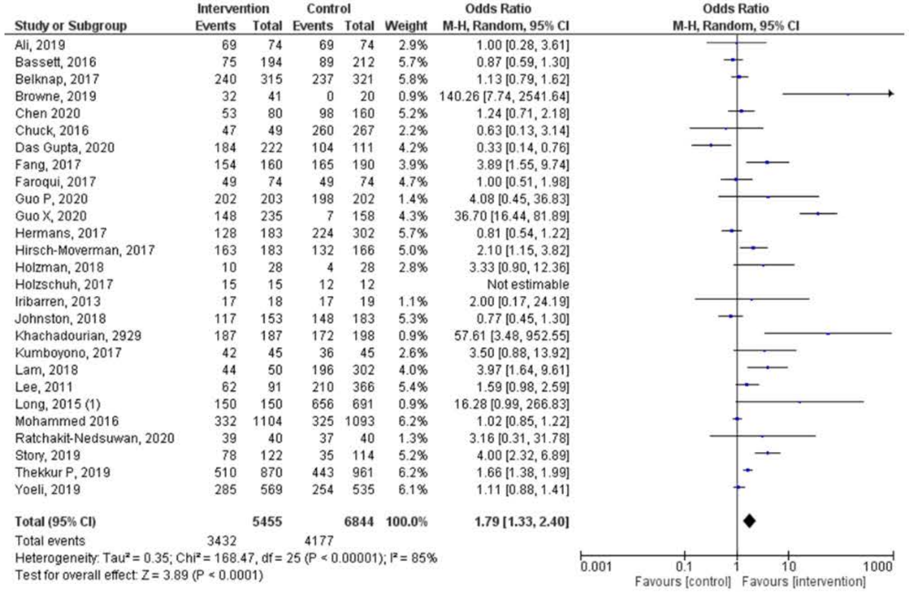

Fig. S2-C: Forest plot of "FEDO" outcome

Study or Subgroup	Intervention Mean	Intervention SD	Intervention Total	Control Mean	Control SD	Control Total	Weight	Mean Difference IV, Random, 95% CI
Bendiksen, 2020	75.9	11.3	11	86.1	26.1	11	9.7%	-10.20 [-27.01, 6.61]
Browne, 2019	93	5.9	41	63	8.1	20	13.0%	30.00 [26.02, 33.98]
DeMaio, 2001	95	3.3	6	97.5	1.7	6	13.1%	-2.50 [-5.47, 0.47]
Garfein, 2018	93	10.1	272	66.4	25.3	272	13.1%	26.60 [23.36, 29.84]
Holzman, 2018	72	18.5	28	66	7.4	28	12.4%	6.00 [-1.38, 13.38]
Johnston, 2018	100	2.2	153	100	2.2	183	13.3%	0.00 [-0.47, 0.47]
Story, 2019	86	17	112	73	27	114	12.7%	13.00 [7.13, 18.87]
Wade, 2012	87.9	19.5397	58	68.9	19.5397	70	12.5%	19.00 [12.20, 25.80]
**Total (95% CI)**			**681**			**704**	**100.0%**	**10.86 [0.75, 20.97]**

Heterogeneity: $Tau^2 = 200.00$; $Chi^2 = 512.82$, df = 7 ($P < 0.00001$); $I^2 = 99\%$
Test for overall effect: $Z = 2.11$ ($P = 0.04$)

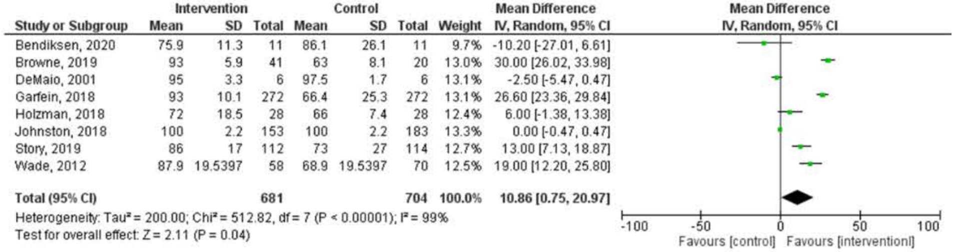

Fig. S2-D: Forest plot of "Cure rate" outcome

Study or Subgroup	Intervention Events	Intervention Total	Control Events	Control Total	Weight	Odds Ratio M-H, Random, 95% CI
Ali, 2019	58	74	44	74	9.6%	2.47 [1.20, 5.09]
Bediang, 2018	87	137	88	142	14.3%	1.07 [0.66, 1.74]
Faroqui, 2017	21	31	20	34	6.1%	1.47 [0.53, 4.06]
Guo P, 2020	186	203	177	202	10.9%	1.55 [0.81, 2.96]
Lee, 2011	9	91	31	366	8.8%	1.19 [0.54, 2.59]
Long, 2015 (1)	43	50	99	187	7.9%	5.46 [2.34, 12.76]
Mohammed 2016	585	1104	578	1093	22.0%	1.00 [0.85, 1.19]
Thekkur P, 2019	95	870	293	961		Not estimable
Yoeli, 2019	260	569	211	535	20.4%	1.29 [1.02, 1.64]
**Total (95% CI)**		**2259**		**2633**	**100.0%**	**1.45 [1.08, 1.94]**
Total events	1249		1248			

Heterogeneity: $Tau^2 = 0.09$; $Chi^2 = 21.70$, df = 7 ($P = 0.003$); $I^2 = 68\%$
Test for overall effect: $Z = 2.49$ ($P = 0.01$)

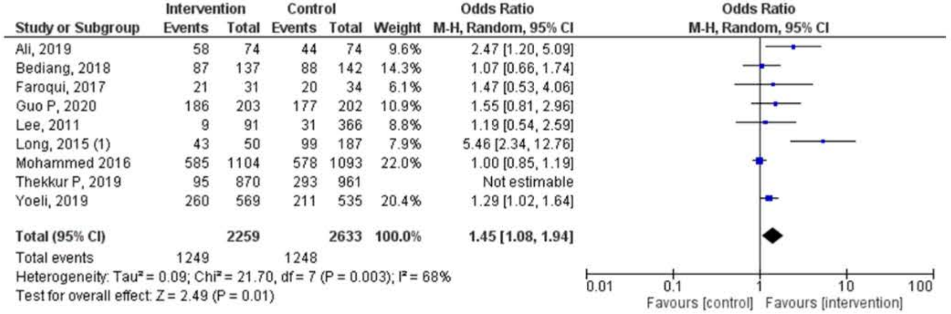

Fig. S2-E: Forest plot of "TIme saving" outcome

Study or Subgroup	Intervention Mean	Intervention SD	Intervention Total	Control Mean	Control SD	Control Total	Weight	Mean Difference IV, Random, 95% CI
Beeler Asay, 2020	4.9	1.8	399	10.3	4.8	740	13.0%	-5.40 [-5.79, -5.01]
Beeler Asay, 2020	4.9	1.8	399	16.7	8.1	452	12.9%	-11.80 [-12.57, -11.03]
Beeler Asay, 2020	5.6	2.7	257	10.3	4.8	740	13.0%	-4.70 [-5.18, -4.22]
Beeler Asay, 2020	5.6	2.7	257	16.7	8.1	452	12.9%	-11.10 [-11.92, -10.28]
Bendiksen, 2020	3	1.8	17	17	9.5	17	9.4%	-14.00 [-18.60, -9.40]
Guo P, 2020	27.6	16.5	203	44.1	32.7	202	8.9%	-16.50 [-21.55, -11.45]
Ha, 2016	2.8	2	266	5	3	278	13.0%	-2.20 [-2.63, -1.77]
Story, 2019	3.2	0.5	122	56	54	114	4.7%	-52.80 [-62.71, -42.89]
Story, 2019	3.2	0.5	122	15	12	144	12.2%	-11.80 [-13.76, -9.84]
**Total (95% CI)**			**2042**			**3139**	**100.0%**	**-11.25 [-13.92, -8.57]**

Heterogeneity: $Tau^2 = 14.28$; $Chi^2 = 838.81$, df = 8 ($P < 0.00001$); $I^2 = 99\%$
Test for overall effect: $Z = 8.24$ ($P < 0.00001$)

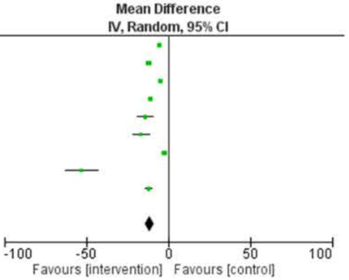

I^2 values >= 50% indicate study heterogeneity is high, hence random effects model was chosen as per recommendations. Forest plots show a tendency favouring interventions. For S2-A an event was defined as a person being correctly referred for TB diagnosis. For S2-B an event was defined as a patient reported as completed TB treatment. For S2-C, the number of reported missed doses and the number of patients in the

study were analysed in order to calculate the mean difference of missed reported doses between groups. For S2-D, an event was defined as a patient reported as cured within a study. For S2-E the time (in minutes) necessary to conduct a consultation or administer a dose of observed medication was input in the meta-analysis in order to analyse the mean difference of time spent between intervention and standard of care groups.

Supplementary Figure S3: Meta-analysis, funnel plots

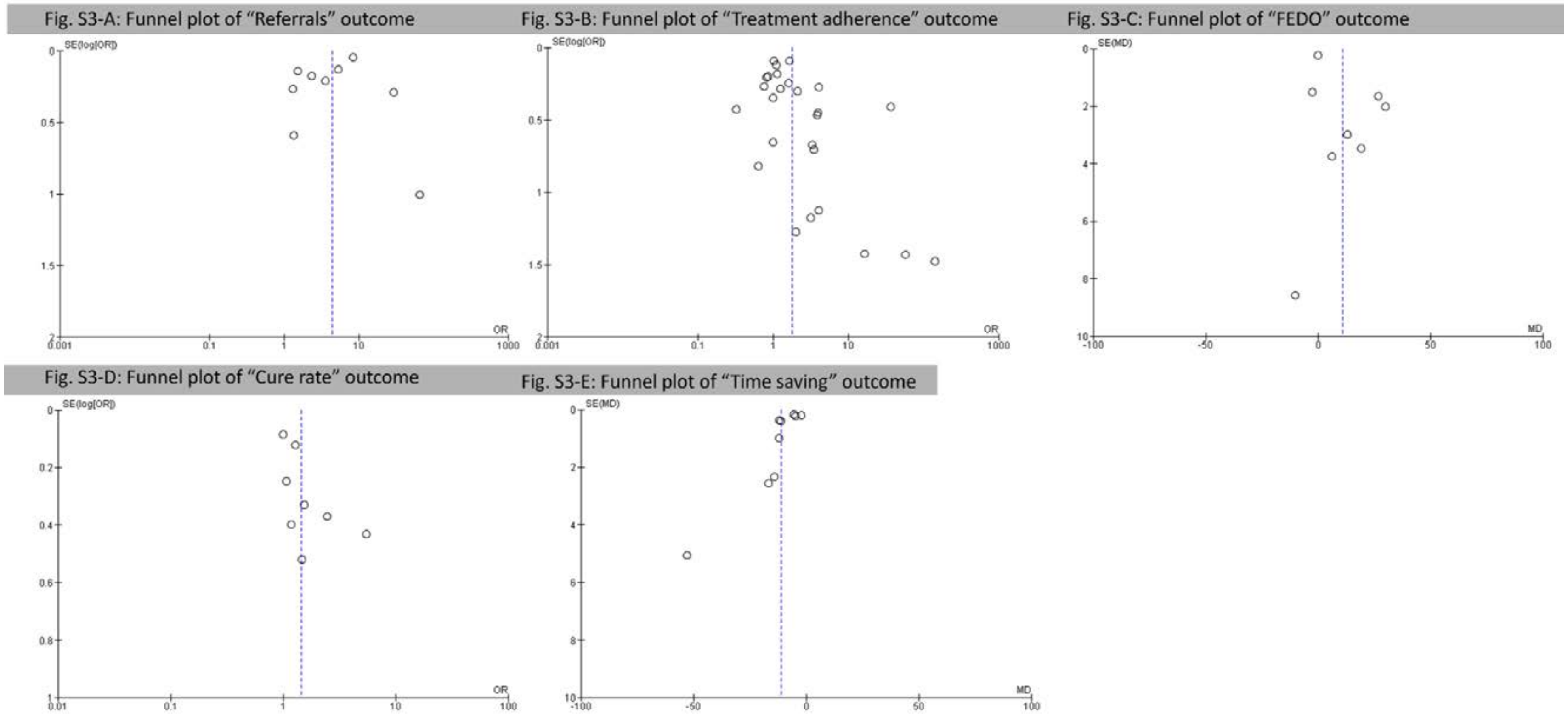

Funnel plots were used to visually analyse the possibility of publication bias. Funnel plots are especially useful when meta-analysis contains smaller studies as they tend to show larger effect sizes and greater variability. The X-axis represents effect estimates and the Y-axis study

precision (study size). Funnel plots were analysed visually for symmetry and potential sources of bias for outliers were taken into consideration. When results were corroborated with the meta-analysis, the sources of bias were better explained by study designs rather than by publication bias. Within all studies, a majority were of smaller scale, and within them, negative results were published and included in the funnel plots, thus, overall, the authors considered that there is a relatively small chance for publication bias.

Supplementary Table S1: Summary of study characteristics

TB = tuberculosis; RCT = randomised control trial; CRCT = cluster randomised control trial; NRCT = non-randomised control trial; BAS = before and after study; SMS = short messaging system.
PLHIV - people living with HIV

First author	Country	Year	Population (P)<br>Intervention (I)<br>Comparison (C)	Type<br>GRADE	Summary of Outcomes
Z.F. Udwadia	India	2021	P: TB patients, 28<br>I: Tele-consults, notifications<br>C: Standard of care, same cohort	BAS<br>1	68% of patients preferred the convenience of intervention; 27% of patients preferred control; 5% of patients had no specific preference.
J. Hodges	Russia	2021	P: TB PLHIV patients, 60<br>I: Notifications, support<br>C: Standard of care, historical cohort	BAS<br>1	Lower mortality by 6 months in the intervention subset (1 death, not attributed to HIV or TB) compared with the pre-intervention cohort (10 deaths, 7 attributed to HIV, p = 0.02).<br>Exposure to the intervention was associated with a decreased likelihood of developing the composite outcome (adjusted odds ratio = 0.33, p = 0.029). Retention of care scale indicators high in intervention.
S.H. Chen	China	2020	P: TB patients, 80 intervention, 160 control<br>I: Video-DOT<br>C: Standard of care	NRCT<br>2	Adherence intervention 66.60% vs control 61.42% (p = 0.001). Satisfaction with location arrangement (p < 0.001), ensuring treatment adherence (p = 0.027) were higher in intervention. Satisfaction with privacy issues (p = 0.005) were superior in the intervention group.
J.N. Sekandi	Uganda	2020	P: TB patients, 50<br>I: Video-DOT<br>C: Standard of care, same cohort	BAS<br>1	82.2% of expected videos were received. The median fraction of expected doses observed was 85%. 98% were satisfied and 88% found the intervention easy to use. Phone malfunction, dead batteries, app errors, network and electricity disruptions accounted for lost videos.
P. Rajpurkar	South Africa	2020	P: Prospective TB patients, 114<br>I: Automated diagnosis based on radiology and clinical parameters (CheXaid)<br>C: Standard of care, same cohort	BAS<br>2	The stand-alone algorithm: mean accuracy of 0.79 (95% CI 0.77, 0.82) vs physicians: mean assisted accuracy of 0.65 (95% CI 0.60, 0.70). The stand-alone algorithm had a sensitivity of 0.67 (95% CI 0.62, 0.73) and specificity of 0.87 (95% CI 0.85, 0.90).
L. DiAndreth	South Africa	2020	P: Prospective TB/HIV+ patients, intervention 226; control 174; Survey respondents: 159<br>I: Diagnosis notification system via SMS/calls<br>C: Standard of Care	NRCT<br>2	Viewed their test results within 7 days of their enrollment: intervention (73.0%) vs control 8.6% (p < 0.001); likelihood to return to clinic: intervention 20.0% vs control 8.6% (p=0.02). 95% felt their information was more protected and confidential when delivered via phone than by the clinic; 96.9% wanted to receive other health information on their phone; 96.9% preferred intervention. No participant reported intervention causing accidental disclosures or instances where others saw their laboratory results without permission.
F.A. Khan	Pakistan	2020	P: Prospective TB patients, 2198<br>I: Radiology diagnosis aid (qXRv2 & CAD4TBv6)<br>C: Standard of care, same cohort	BAS<br>2	qXRv2, overall sensitivity was 0·93 (95% CI 0·89–0·95) and specificity was 0·75 (0·73–0·77). CAD4TBv6 sensitivity (0·93, 0·90–0·96) had specificity 0·69 (0·67–0·71).
G.R. Beeler Asay	USA	2020	P: TB patients, intervention 103; control 122; Sessions: intervention 173; control 170	BAS<br>2	Live Video-DOT time 4.86 minutes (95% CI = 3.77,6.26) vs recorded Video-DOT 5.62 (95% CI = 4.06,7.77) vs clinic DOT 10.27 (95% CI = 7.51, 14.04) vs field DOT 10.13 (95% CI = 7.89, 3.01) + travel

			I: Video-DOT synchronous and asynchronous<br>C: Standard of care, historical cohort		time 16.67 minutes (95% CI = 12.08, 22.99). Costs per session are lower in Video-DOT than DOT groups for healthcare and lower in Video-DOT also for patients.
P. Guo	China	2020	P: TB patients, intervention 203, control 202; Survey respondents<br>Intervention 199, Control 196<br>I: Video-DOT asynchronous<br>C: Standard of care	RCT<br>4	Cured: intervention 186 (91.6%) vs control 177 (87.6%); lost to follow up intervention 1 (0.5%) vs control 4 (2.0%) (not statistically significant). Average time per dose observed intervention 16.5 min vs control 44.1 DOT (including travel time), p < 0.01. Costs intervention Y-- 34.3 (4.41 EUR) vs control Y-- 71.6 (9.21 EUR), p < 0.01. Survey responses: “convenient & comfortable” intervention 191 [96.0%] vs. control 111 [56.6%], p < 0.001, “would recommend the method to other patients” intervention 191 [96.0%] vs. control 113 [57.7%], p < 0.001.
L. Ravenscroft	Rep. of Moldova	2020	P: TB patients, intervention: 85, control 90<br>I: Video-DOT, asynchronous<br>C: Standard of care	RCT<br>4	Failure to adhere: control 5.24 days/14 days vs intervention 1.29 days/14 days, (95% CI 3.35–4.67 days, p<0.01). Intervention saved an average of 58 h (95% CI 48–68 h; p<0.01) and an average EUR 25 over the course of the 4-month study period (self-reported). Cumulative log-odds increment in satisfaction of being in the intervention treatment group is 3.29 (95% CI 1.66–4.92; p<0.01). Treatment success not statistically different. Intervention patients are 11% more likely to report side-effects.
X. Guo	China	2020	P: TB patients, intervention 235; control 158; Survey respondents: intervention 235; control 131; Medical staff 66<br>I: Video-DOT synchronous, reminders<br>C: Standard of care	NRCT<br>2	≥95% of doses observed: intervention 63.0% (148/235) vs control: 4.4% (7/158) (P<.001). 5 patients in the intervention group failed to send a video for at least three consecutive days and were advised to transfer to control, but all of them requested to be allowed to remain on intervention. The median estimated total time spent traveling over 6 months: control 60 hours vs intervention 12 hours.<br>Estimated total transportation costs: control intervention ¥720 vs control ¥96 (difference of 25 EUR) Patients satisfied: intervention 191/235 (81%) vs control 53/131 (40%). Felt there was no violation in privacy: intervention 133/235 (57%), control 85/131 (65%). Most medical staff preferred intervention.
F. Madhani	Pakistan	2020	P: Prospective TB patients: 127 062<br>I: Radiology diagnosis aid (CAD4TB)<br>C: Standard of care, same cohort	BAS<br>2	Prevalence of Xpert positivity was lowest (0.7%) in the under 50 category (CAD4TB score), and rose to 23.5% in the over 90 category overall, and the trend was similar in both hospital and community settings.
D. Das Gupta	India	2020	P: TB patients, intervention 222, control 111; Medical staff: 8<br>I: SMS and phone reminders<br>C: Standard of care	NRCT<br>1	Treatment completion: intervention group A: 93/111 (84%), group B: 91/111 (82%), control: 104/111(93%) (not significant). Reminder cues contributed to an increase in their self-motivation, the interviewed patients also indicated feeling that they had received personal care, with someone always there to remind them to take their medicine on a regular basis.
V. Khachadouri an	Armenia	2020	P: TB patients, intervention 187; control 198<br>I: Counselling; SMS and phone reminders<br>C: Standard of care	cRCT<br>3	Adherence: intervention 100% vs Control 87.3% patients. Intervention 78.4% reported that the text messages were helpful in reminding them to take their drugs; 66.2% to visit the clinic weekly; 10% would have taken the drugs regardless of those reminders. 80.9% of family supporters reported that phone calls helped them feel confident that the disease was under control.
S.S. Habib	Pakistan	2020	P: Diabetes mellitus potential TB patients: 694<br>I: Radiology diagnosis aid (CAD4TB)<br>C: Standard of care, same cohort	BAS<br>2	Intervention cut-offs (automated score) of 50 and 90 yielded sensitivities of 90.5% and 48.7% respectively. Potential TB cases missed and Xpert testing yield were the lowest at the cut-off of 50 and highest at 90.

K. Murphy	Pakistan	2020	P: X-Rays: 5565; compared to experts: 500<br>I: Radiology diagnosis aid (CAD4TB)<br>C: Standard of care, cohort	BAS<br>2	With sensitivity set at 90% the system can achieve 76% specificity. The performance of CAD4TB v6 is very similar to expert observers, particularly at high sensitivities, and no observer is seen to perform significantly better (above the 95% confidence interval) than CAD4TB v6 at any operating point. Cost savings per TB case detected at 0.95 sensitivity is 36.18 EUR.
R. Ratchakit-Nedsuwan	Thailand	2020	P: TB patients initial, intervention 50, control 50; month 6, intervention 40, control 40; focus group 8<br>I: Pill box, reminders, calls<br>C: Standard of care	RCT<br>4	>80% of doses: control 37/40 vs intervention 39/40, hazard ratio 3.2 (0.2–170.2). Treatment success: control 39/41 vs intervention 36/40 (no significant difference). Most of the participants reported positive feedback regarding the core functions of the intervention. Half of the participants experienced lack of or a poor mobile network signal and fast battery draining. A few participants commented about the size and weight of the device, too-small instruction font size on the lid, an inaudible alarm volume when placing it on another floor of the house, and concern about small children's reactions.
M. Nash	India	2020	P: X-Rays, intervention 317; control 612<br>I: Radiology diagnosis aid (qXR)<br>C: Standard of care, same cohort	NRCT<br>2	For the general classification of an X-Ray as 'abnormal', intervention AUC of 0.87 (95% CI: 0.84, 0.91). The lowest AUC achieved by intervention, 0.75 (95% CI 0.70, 0.80) and 0.76 (95% CI: 0.73, 0.79), were for detection of 'hilar lymphadenopathy' and 'consolidation', respectively. For detecting abnormalities 'cavity,' 'fibrosis,' 'pleural effusion', 'opacity' and 'blunted costophrenic angle', intervention achieved AUC ranging from 0.84 to 0.94. The highest AUC achieved by qXR, 0.94 (95% CI: 0.91, 0.96), was for detection of 'cardiomegaly'.
R. Bendiksen	Norway	2020	P: TB patients: 17; Medical staff: 17<br>I: Video-DOT, synchronous<br>C: Standard of care, same cohort	BAS<br>1	Compliance with medication intake: intervention 89.8% vs control 95.4%. The median time spent by medical staff: control 17 (2–40) minutes vs intervention 3 (1–8) minutes. Medical staff opinions about frequency of practical problems in intervention: 5 "never", 7 "rarely", 5 "often". Technical problems (8.9%, 268 out of 3,023 days during the intervention) were the most common single reason why intervention was not performed. Patients' opinion about confidentiality 11 "better in intervention", 6 "I do not know". 14 out of 17 patients preferred intervention, and 15 would recommend intervention to others.14 out of 17 medical staff preferred intervention and all wanted I to be continued for new patients.
A. Prabhu	India	2020	P: TB-PLHIV patients 72; Medical staff 21<br>I: Pill box with phone number for adherence<br>C: Standard of care, same cohort	BAS<br>1	Average adherence:intervention 27% vs treatment card 99% in the TB treatment card (p < 0.0001). Treatment completion 49 of 72 (68%), death 15 (21%), loss to follow up 3 (4%). Primary issue was missed calls not being registered (app error). Medical staff challenge was Lack of communication between medical staff. Patient challenges: lack of motivation, confusion regarding procedures, not owning a mobile phone or sharing.
E. Yoeli	Kenya	2019	P: TB patients: Intervention 569, Control 535<br>I: SMS and calls for adherence. Gamification element.<br>C: Standard of care	RCT<br>3	Unsuccessful treatment outcomes: intervention 24 patients (4.2%) vs control 70 patients (13.1%) (P<0.001).
S.B. Holzman	India	2019	P: TB patients, 25; survey respondents, 22<br>I: Video-DOT, asynchronous, SMS reminders<br>C: Standard of care, same cohort	BAS<br>1	Median adherence on vDOT was 74% (IQR 62%-84%). After including verbally verified doses (following unverifiable or incomplete videos), the median verifiable fraction was 86% (IQR 74%-98%). A total of 91% (20/22) of surveyed patients described intervention as easy to use. 91% (20/22) found text message reminders helpful. The majority felt intervention would be more convenient (20/22, 91%) and preferred (20/22, 91%) over in-person DOT. 82% (18/22) felt intervention would preserve patient privacy over in-person DOT, 18% (4/22) disagreed.
N. Wang	China	2019	P: TB patients, 169 (15 switched to standard of care); Medical staff, 9	BAS<br>1	Median adherence rate (average percentage of doses taken) was 99.3% (83.4% - 100.0%). Factors increasing the likelihood of patients switching back to standard of care: migrants vs local persons,

			I: Pill Box<br>C: Standard of care, historical cohort		retreatment vs new TB cases, over 65 years of age. Medical staff agreed intervention was useful. 8/9 considered the intervention to be a moderate increase in their workloads. Number of visits in standard of care 38,160 vs intervention 4604 (decrease of 87.9%).
C.K. Lam	USA	2019	P: DOT Sessions: 38 035, clinic DOT: 12002; field DOT: 15483; live VDOT: 7185; asynchronous VDOT: 3365<br>I: Video-DOT synchronous and asynchronous<br>C: Standard of care, historical cohort	BAS 2	Total cost per session (labor + non-labor): I live-VDOT 6.54, I recorded-VDOT 5.35, C clinic-DOT 8.46, C field-DOT 19.83. Total annual DOT cost (247 working days): I live-VDOT 46 927, I recorded-VDOT 18 463, C clinic-DOT 102, C field-DOT 494 308 521. Increasing I in the future would reduce costs.
J.E. Farley	South Africa	2019	P: Prospective TB patients 6341; Resistant-TB patients: 41<br>I: Notification system for medical staff and patients<br>C: Standard of care, historical cohort	BAS 1	Intervention time from diagnosis to linkage to care: 10h.41 mins. Time from linkage to care to treatment initiation: 2 days. 11h.31 min; total 3 days, 21 h, 17 min vs control 10 -22 days. Intervention 5 (12%) lost to follow-up vs control 30%.
R.H.H.M. Philipsen	Philippines	2019	P: X-Rays: 10 755 of which 200 were used with independent expert<br>I: Radiology diagnosis aid (CAD4TB)<br>C: Standard of care, same cohort	BAS 2	Using a threshold of 60, the software had a sensitivity of 0.98 and a specificity of 0.69. For the random 200, the physician had a sensitivity of 0.82 (95%CI 0.74–0.89) and specificity of 0.87 (95%CI 0.81–0.96) vs software: same specificity and not statistically different slightly higher sensitivity (0.83, 95%CI 0.72–0.94; P ¼ 0.739).
S.H. Browne	South Africa	2019	P: TB patients intervention 41, control 21<br>I: Ingested medication monitor<br>C: Standard of care	RCT 4	Intent-to-treat (ITT) analysis within the RCT showed intervention confirmed 93% versus 63% control (p < 0.001) of daily doses prescribed. 100% of participants preferred using intervention.
A.O.A. Ali	Sudan	2019	P: TB Patients: Intervention 74; control: 74<br>I: Reminder SMS and calls during treatment.<br>C: Standard of care	NRCT 2	Default rate: Intervention 6.8%; 5 out of 74 vs control: 10.8%; 8 out of 74 (P-value 0.563; OR: 1.673, 95% C.I. 0.521- 5.374). Cure rate Intervention 58 /74 (78.4.0%) vs control: 44 (59.5) of the 74 (P-value 0.020; OR: 2,472, 95% CI:1.133 – 5.434). At the end of treatment, the knowledge in the intervention group was better than in the control group. Intervention rated as “useful”: 72(97.3%) and “not useful” 2 (2.7%).
S. Palupi	Indonesia	2019	P: TB samples: Intervention 2479, Control 9<br>I: notification system<br>C: Standard of care	BAS 1	Specimen referral went up from 9 cases referred for rapid molecular testing (RMT) in sept 2017 to 2479 in sept 2018 (after intervention). Notification of results “within a minute”.
J. L. Davis	Uganda	2019	P: TB Patients Intervention 190; Control 213; TB Contacts Intervention 471; Control 448<br>I: SMS reminders, notification system<br>C: Standard of care	RCT 3	SMS delivered to 95 (50%) of the intervention arm (programming error).<br>Yield of contact investigation Intervention 7/471, 1.5% vs control 5/448, 1.1%(OR 1.34, 95% CI 0.42–4.24, p=0.62). <20% of SMSs achieved their full effects. Barriers to intervention: sharing phones, broken phones, inability to read text messages, lack of familiarity with or attentiveness to SMS, preference for in-person disclosure of results.
S. Moayedi-Nia	Canada	2019	P: Tuberculin skin tests: 64 photos of administration, 72 photos of induration; Medical staff: 6<br>I: Automated photo analysis of TST on mobile phone app	BAS 2	TST induration, proportion of “correct on first reading”: 0-4mm intervention 95% vs control 83%, 5-9mm, intervention 20% vs control 33%, 10-14mm intervention 77% vs control 67%, >=15mm intervention 92% vs control 91%.

			C: Standard of care, same cohort		
P. Thekkur	India	2019	P: TB PLHIV+ Patients, intervention 870, control 961<br>I: Phone call reminders and automated answer after medication administration<br>C: Standard of care	NRCT<br>1	Successful intervention 605 (69.5) vs control 736 (76.6) <p 0.001. Poor implementation of intervention meant that treatment was mostly unsupervised. Treatment completed intervention 510/870 (58.6%) vs control 443/961 (46.1%) p < 0.001. Challenges: not owning a mobile phone, not knowing how to use, lack of motivation, app-related challenges, preference for human interaction, staff related issues.
A. Story	UK	2019	P: TB Patients: Intervention 114, Control 112<br>I: Video-DOT asynchronous<br>C: Standard of care	RCT<br>4	≥80% scheduled observations successfully completed during the first 2 months:<br>70% I vs 31% C (adjusted odds ratio [OR] 5·48, 95% CI 3·10–9·68; p<0·0001).<br>Average staff time per dose observed was C 56 min community based observed therapy; C 15 min for clinic-based observed therapy, and 3·2 min I. The costs C £5700 per patient vs I £1645 per patient.
A. Wagstaff	South Africa	2019	P: Potential TB patients: intervention group A163, group B 155, control 97<br>I: SMS reminders<br>C: Standard of care	RCT<br>4	Returned to collect TB diagnosis results: 78/101 C vs 331/405 I. HIV patients were more responsive to the SMS. Non-delivery of the message excluded 15% of initial I participants and human error 5%.
K. Schwab	Malawi	2018	P: Potential TB cases: 181 examinations, in-depth analysis 108,1629 images; Medical staff: 11<br>I: Tele-ultrasound<br>C: Standard of care, same cohort	BAS<br>1	General labelling of images as abnormal: intervention 96 (6%) vs control 85 (5%) as abnormal, revealing an overall agreement of 98%. Pericardial effusion: 99.1%, periportal LAD 98.1%, para-aortic LAD 98.1%, left pleural effusion 99.1%, right pleural effusion 95.4%, ascites 99.1%, liver lesions 99.1%, splenic lesions 99.1%, other abnormalities 93.5%, any abnormality 97.8% Clinicians identified 92% of abnormalities seen by the expert reader.
R. S. Garfein	USA	2018	P: TB Patients Intervention 274; Control 159<br>I: Video-DOT, asynchronous<br>C: Standard of care, historical cohort	BAS<br>2	Fraction of expected doses observed (FEDO): 93% intervention vs 66.4% control. FEDO increased with longer use of intervention, higher annual income, and decreased with marihuana use in the past 6 months, poor network connection, taking medications away from home. Intervention was 6-46% cheaper than observed therapy (range $3,031–$3,911 versus range $3,212–$5,788) mostly due to personnel costs. 90% would choose intervention. Most believed I was more confidential than control.
C. K. Lam	USA	2018	P: LTBI Patients Intervention 50, Control 302<br>I: Video-DOT, synchronous<br>C: Standard of care, historical cohort, same cohort	BAS<br>2	205 issues, of which: health department related 29, patient equipment 43, patient knowledge 3. Technical issues did not prevent continuation of observation sessions. Completed treatment intervention 44 (88.0%) vs control 196 (64.9%).
N. Maraba	South Africa	2018	P: TB patients, intervention 319, control 457; TB patients Interviewed: 14; Medical staff: 7 interviewed<br>I: notification system<br>C: Standard of care, same cohort	BAS<br>1	Proportion of results available within 48 hours: I 96.8% vs C 68.6% (p <0.001).<br>Proportion of treatment within 28 days I 28/33 (84.8%) vs C 30/44 (68.2%), (p = 0.08). In-depth interviews showed that providers easily integrated the intervention application into routine TB investigation and patients positively received the delivery of results via text message. Time from sputum collection to TB treatment initiation 4 days control vs to 3 days, not statistically significant.
S. B. Holzman	USA	2018	P: TB patients: 28<br>I: VDOT asynchronous<br>C: Standard of care, same cohort	BAS<br>1	Adherence: I 94% vs C 98%, P = .17). Total treatment doses observed: I 72% vs C 66%, P = .03. Staff, patients: cited increased treatment flexibility, convenience, and patient privacy in I. Cost analysis estimates savings with I of $1391 per patient for a standard 6-month treatment course.

G.Bediang	Cameroon	2018	P: TB patients, intervention 137, control 142<br>I: SMS reminders<br>C: Standard of care	RCT<br>3	At 6 months: cure rate 87 (63.5%) in I vs 88 (62%) in C (OR = 1.06 [0.65, 1.73]; p = 0.791). Satisfaction general management: 99.5 I and 99.2% C (p = 0.41).” Support provided for adherence to drug prescriptions - 99.6% of satisfaction in I vs 99.1% in C (MD: 0.5% [− 0.2, 1.2]; p = 0.1).
J. C. Johnston	Canada	2018	P: LTBI patients, intervention 170, control 188<br>I: SMS reminders<br>C: Standard of Care	RCT<br>4	Intention-to-treat analysis, proportion of participants completing LTBI therapy: 79.4% I and 81.9% C, (RR 0.97, 95% CI 0.88–1.07; p=0.550). Results were similar for pre-specified secondary endpoints, including time-to-completion of LTBI therapy, completion of >90% of prescribed LTBI doses and health-related quality of life.
R. Belknap	USA, Spain, Hong Kong, South Africa	2018	P: TB patients, intervention 315, control 321<br>I: SMS reminders<br>C: Standard of Care	RCT<br>3	Treatment completion: 76.4% (CI, 71.3% - 80.8%) intervention vs 74.0% (CI, 68.9% - 78.6%) control.
T. Buchman	USA	2017	P: TB patients, intervention 24, control 94<br>I: Video-DOT synchronous<br>C: Standard of care, same cohort	BAS<br>1	Total mileage savings and time were $9,929.07 and 614 hours.
J. Gao	China Canada	2018	P: Participants, intervention 193, control 134<br>I: Educational video<br>C: Standard of care, same cohort	BAS<br>2	Viewing the video was associated with a 1.04 (95% CI 0.85–1.26) or a 21% increase in a knowledge score. Of 193 viewers who completed the survey, 84% rated the TB video as “somewhat/very helpful”, and 89% might recommend the video to others.”
Y. Hirsch-Moverman	Lesotho	2017	P: TB PLHIV+ patients, intervention 183, control 166; Interviewees, patients 30, medical staff: 30<br>I: SMS and phone call reminders<br>C: Standard of care	CRCT<br>2	41.9% stated that intervention increased adherence; 89.1% in I vs 79.5% in control. Facilitators: cues for medication & appointments, access to telephone; choice in selecting time & frequency of intervention. Challenges: lack of previous phone usage, coded messages were confusing, electricity, technical. Satisfaction: medical staff expressed support; patients were appreciative.
J. Strymish	USA	2017	P: Prospective LTBI Patients, intervention 285 (39 LTBI), control 195<br>I: Tele-consultation<br>C: Standard of care	NRCT<br>1	Intervention is faster: 0.6 days (SD 3.6) vs 16.5 days (SD 12.4) P < .0; Intervention expands volume of consults without reducing the number of time per consults: 285 intervention vs 195 control. Intervention is most useful for patients who need to travel long distances to reach the clinic.
R. J. Farooqi	Pakistan	2017	P: TB patients, intervention 74, control 74<br>I: SMS reminders<br>C: Standard of care	RCT<br>2	Outcomes were not significantly different: 21 cured in intervention vs 20 in control.
S.M. Hermans	Uganda	2017	P: TB PLHIV+ patients: Intervention 171, Control 274<br>I: SMS and phone call reminders<br>C: Standard of care, historical cohort	NRCT<br>1	Composite outcome 8 weeks of treatment & end of treatment: not significantly different. Increase: 6/8 test questions baseline to 7/8 test questions. 165 (96%) rated intervention as helpful or very helpful; 92% intervention was helpful. 0 breaches of confidentiality. 28% did not understand the message, 26% unable to use phone for a mean duration of 14 (IQR 5-28) days; Main challenges: network, provider, system did not recognise reply; human-related issues.

K. Choun	Cambodia	2017	P: TB patients, 106<br>I: Phone reminders, mobile app<br>C: Standard of care, historical cohort	BAS<br>2	Intervention 103 (97%) contacted & placed/continued on TB treatment vs control 31-81%. Facilitators: patients not traced directly were traced through health facilities.
S. Chadha	India	2017	P: Medical staff, intervention 30, control139; Potential TB cases, intervention 1056, control 552; TB patients diagnosed, intervention 127, control 67; Diagnosed on day 1, intervention 99, control 59<br>I: Notification system<br>C: Standard of care	NRCT<br>2	The number of TB patients diagnosed on day 1 (day of referral) was 158/194 (82%); 99/194 (51%) were referred by intervention. The number of diagnosed TB patients started on treatment on day 1 was 80/194 (41%); 48/80 (60%) were referred by intervention. The remaining patients were started on treatment within 7 days of diagnosis. The number of patients who received observed therapy: 60 from intervention and 39 from control.
X.H. Fang	China	2017	P: TB patients Intervention 160; Control 190<br>I: SMS reminders<br>C: Standard of care	CRCT<br>3	Re-examined sputum 2 months intervention 96.88% vs. control 87.89%, p=0.002, 6 months Intervention 88.13% vs. control 69.47%, p=0.001. Completed the treatment intervention 154 (96.25%) vs. control 165 (86.84%), p=0.002.
E. L. Holzschuh	USA	2017	P: TB patients: Intervention 15, Control 12<br>I: Video-DOT asynchronous<br>C: Standard of care, same cohort	NRCT<br>1	14 completed treatment; 1 interrupted - adverse event. $2,066 saved in km.<br>Advantageous in case of travel, family relocation.
I. Prieto-Egido	Spain	2016	P: TB Diagnosis Samples: 70 sputum and 20 bronchial aspirate<br>I: Tele-microbiology<br>C: Standard of care, same cohort	BAS<br>1	100% concordance between intervention and control. Intervention took 7x longer than control - field of vision 10x smaller; remote interaction could be a challenge.
C.Chuck	USA	2016	P: TB patients, intervention 49, control 267<br>I: Video-DOT synchronous<br>C: Standard of care, same cohort	BAS<br>2	Completed treatment: intervention 47 (96%) vs control 260/267 (97%). Issues reported by 54 patients, 276 were technical problems, 49 were patient-related challenges such as patients forgetting their appointment, having schedule conflicts, or patient being out of camera view; and 21 were due to smartphone misuse. Facilitators: travel, weather, twice capacity.
S. Mohammed	Pakistan	2016	P: TB patients, intervention 1104, control 1093<br>I: SMS reminders, support, confirmation text from patients<br>C: Standard of care	RCT<br>3	Challenges: system failure, administrative, GPRS outage, participants opting out/dying, not knowing their phone number/not sharing; response rates fell in time: 48% in the first two weeks to 24% (eight-month regimen) and 20% (six-month regimen) in the last two weeks. No significant impact on treatment success.
Y. P. Ha	Botswana	2016	P: Medical staff: 2; TB contacts, intervention 89, control 113 TB<br>I: Notification system<br>C: Standard of care	NRCT	Control 5.0 min per contact (IQR 4.0–8.0) vs. intervention 2.8 min per contact (IQR: 1.7–4.4), ($p < .001$); 12/113 (10.6%) contacts had ≥1 missing or illogical values vs 0 in intervention. Intervention proved to be faster, with less errors.. Overall rating 2.1/7.0, system usefulness 1.6/7.0, information quality 2.6/7.0, interface quality 2.3/7.0 (lower scores are better). Challenges: network, system (server).
I. V. Bassett		2016	P: TB patients, intervention 187, control 198	RCT	

	South Africa		I: Counselling, SMS and phone reminders<br>C: Standard of care	4	Proportion of patients completing treatment: 39% intervention vs 42% control; RR 0.93, 95% CI: 0.80 to 1.08).
R. Long	Canada	2015	P: TB patients, intervention 150, control 691<br>I: Virtual clinic<br>C: Standard of care, same cohort	BAS<br>2	6 treatment outcome indicators both groups similar. Followed to three smears negative intervention > control (93% I vs 55% C, p = 0.0001); end-of-initial phase sputum culture and chest radiograph, intervention > control (78% C vs 50% I, p = 0.01; 68% C vs 52% I, p = 0.05, respectively); proportion of TB cases treated with observed therapy intervention > control (100.0% I vs 95.0% C, p = 0.004). 3 indicators, one related to case management, one related to treatment outcome and one related to contact management, intervention > control; on 3 indicators, two related to case management and one related to contact management, control > intervention.
X. Liu	China	2015	P: TB patients, intervention 1008 text msg; 997 medication monitor; 1064 combined; control 1104<br>I: Pill Box, SMS reminders, Medication box reminder<br>C: Standard of care	CRCT<br>4	Measure = at least 20% of doses missed: 29.9% control vs 27.3% in intervention (adjusted mean ratio 0.94, 95% CI 0.71, 1.24). Intervention lower loss to follow-up and occurrence of poor treatment outcome than control. Challenges: minor problems 56.5% intervention & 27.3% combined arm: “These problems included incorrect usage of the phone by the patient (42.0%), network failure (21.1%), and no money on the phone account (14.9%). Problems with the medication monitor or phone were resolved in 88.7% of occurrences.“
Kumboyono	Indonesia	2015	P: TB patients, intervention 45, control 45<br>I: SMS reminders, support<br>C: Standard of Care	NRCT<br>1	Treatment compliance intervention 93.3% vs control 80% P=0.059. Awareness not significantly different (Fishers exact).
S. J. Iribarren	Argentina	2015	P: TB patients, intervention 19, control 18<br>I: SMS reminders, confirmation text from patients<br>C: Standard of care	RCT<br>2	Medical staff all agreed that intervention beneficial; Advantage for “rural or semi-rural settings where access to health care was challenging”. Challenges: network, Internet, electricity, travelling, mobile phone provider; different problems with automated software.
M. Mirsaeidi	USA	2015	P: TB patients, 11<br>I: Video-DOT<br>C: Standard of care	BAS<br>1	7 (88%) of the patients interviewed thought it was an improvement & would recommend. Intervention saved 13495.7 EUR per patient, in travel time and operational costs.
N. Lorent	Cambodia	2014	P: Medical staff: 37; Potential TB cases 315.874 screened, 12.201 with TB symptoms; TB patients 10.301<br>I: Notification system<br>C: Standard of care, historical cohort	BAS<br>1	Median of 3 days vs 7-10 days faster time to obtain lab results after implementing the intervention.
T.R. Schulz	Australia	2014	P: 119 patients of which 49 LTBI<br>I: Tele-consultation<br>C: Standard of care, historical cohort	BAS<br>1	Nearly 500 km of travel and 127 kg of CO(2) production was avoided per consultation. Technical issues were faced in 25% of consultations, most frequently sound problems and connections dropping out. A bandwidth of at least 512 kbps and latency of no more than 300 ms is required.
S. J. Iribarren	Argentina	2013	P: TB patients, intervention 19, control 18<br>I: SMS reminders, confirmation text from patients<br>C: Standard of care	RCT<br>2	Helpfulness Intervention 9/12, all components equally helpful. Patients felt “cared by staff”. Treatment completion: 34/37 (92%) total, 17/18 I; 17/19 C.

J. A. Blaya	Peru	2013	P: Health centers intervention 29, control 49; TB patients: intervention 890, control 781<br>I: Notification system<br>C: Standard of care	CRCT<br>4	Receive results: drug susceptibility tests (DST) median 11 intervention vs. 17 control days, p,0.001 and culture 5 intervention vs. 8 control days, p,0.001; faster culture conversion: 16 days sooner intervention, 20% less than control (p = 0.047); treatment turnaround time did not differ significantly 88 v. 77 days, p = 0.28.
K.H. Chaiyachati	South Africa	2013	P: TB patients, 4, Medical staff, 5<br>I: Notification system<br>C: Standard of care, historical cohort	BAS<br>1	Intervention improved communication & collaboration, nurses felt more included; improved workflow. Satisfaction was high among medical staff. One phone malfunctioned, repaired within one week. Programme was periodically freezing. There were no network issues. Medical staff were sometimes forgetting to use intervention. Travel was saved.
M. A. Gassanov	Canada	2012	P: TB patients 13<br>I: Video-DOT, synchronous<br>C: Standard of care, same cohort	BAS<br>1	Treatment compliance same (~ 98%); Average duration of I = 10 min vs 36 min C; Advantages: high flexibility, privacy. Disadvantages: interpersonal connection, few patients had technical issues - individual network.
V. A. Wade	Australia	2012	P: TB patients, intervention 58, control 70; TB Patients Interviewed: 12; Medical staff: 18<br>I: Video-DOT synchronous<br>C: Standard of care	NRCT<br>1	Intervention significantly reduced the percentage of missed observation episodes; non-adherence the same in intervention & control; felt it increases adherence; Benefits: flexibility, convenience; good relationship with nurses; nurses said it's faster; cohesion of medical staff team. 10/12 very satisfied, 2 mixed feelings. Average of 2 days was lost from the Intervention technical, network, electricity, hardware; 1 would prefer control because privacy, but impractical. "If one is willing to pay $2, the probability of cost-effectiveness rises to almost 90%".
R. M. Coulborn	Malawi	2012	P: X-Rays, 159<br>I: Tele-radiology<br>C: Standard of care, same cohort	BAS<br>1	70.9% intervention (radiologist using eHealth) coincided with at least 1 diagnosis in control (clinical staff). Intervention management initially proposed by control 36 patients (23.5%); some had tuberculosis and others had other pulmonary ailments. 2 (1.3%) diagnosed by intervention, missed in control. 1 misdiagnosis of TB corrected by intervention, I averted inappropriate treatment in 16 patients (10.5%).
A. J. Khan	Pakistan	2012	P: Potential TB cases: 469 896 individuals screened, suspected: 7463<br>I: Mobile app for TB screening<br>C: Standard of care, historical cohort	NRCT<br>1	Notification of adult pulmonary tuberculosis increased 3.77 times (415 vs 1576) and childhood pulmonary tuberculosis by 7.32 times (28 vs 205). This was a 2·21 times increase (95% CI 1.93–2.53; p=0.000) relative to the change in the control area, where the number decreased by 9%. Facilitated by: community laypeople and financial incentives.
P. Kunawararak	Thailand	2011	P: TB patients non-MDR intervention 30; control 30; MDR-TB intervention 19, control 19<br>I: Phone call reminder<br>C: Standard of care	NRCT<br>1	MDR: Sputum conversion rate at 1 mo 90% intervention vs 20% control (p<0.001); non MDR: I 37% I and 52% control (p=0.221); MDR and non MDR success rates 100% intervention vs control MDR 73.7% (p=0.0001), non MDR-TB 96.7% (p=0.047). Accidental reveal of disease in control group. Opinion: increased patient communication, awareness, confidentiality.
T.C. Chen	Taiwan/China	2011	P: TB patients, intervention 127, control 96<br>I: Notification system, reminders<br>C: Standard of care	NRCT<br>2	Laboratory delay 3 control vs 1 intervention days (P<.001), response delay 0 control vs 0 intervention (P<.045), interval from admission to transfer to the isolation room 8.5 control vs 3 intervention days (P <.001). Proportion of patients transferred to isolation within 1 day increased significantly. No significant difference in the total number of medical staff exposed to each active TB patient in control vs intervention, but reported number of nurses exposed per patient with active TB who stayed in the general ward was significantly less during the intervention phase (P<.039).
A. Marcelo	Pakistan	2011	P: TB patients, 88<br>I: Tele-radiology	BAS<br>1	Agreement in 71 cases (80.6%) and disagreement in 17 cases (19.38%) between intervention and control. Average delay in waiting for results, less with 34.6 hours (range 9 minutes to 289.2 hours) after

			C: Standard of care, same cohort		intervention. The average delay at the rural site (59.15 hours) was more than for the urban site (15.9 hours). Culture results: intervention 32.4% positive vs control 27.6% positive. 2-month clinical follow-up: better improvement in symptoms and weight of the patients diagnosed in control vs intervention. Medical staff showed satisfaction with the quality of images, were at complete ease in making diagnostic decisions, and never requested repeat Xrays.
J. A. Blaya	Peru	2011	P: Medical staff intervention 891, control 780; Smear Microscopy Intervention 1623, control 1348; Culture intervention 4203, control 2130<br>I: Notification system<br>C: Standard of care	CRCT<br>4	Faster to receive results: drug susceptibility tests (DST) median 9 vs 16 days, p<0.001 and culture results 4 vs 8 days, p<0.001; peripheral health centres, communication times for DST (median 22 I vs 19 C days, p<0.30) and culture (10 intervention vs 9 control days, p¼0.10) results, same proportion of 'late' DSTs (p<0.57) compared with the control. Intervention does not "trickle down" to peripheral health centres.
J. E. Lee	Korea	2010	P: TB patients, intervention 163, control 441<br>I: Notification system<br>C: Standard of care, historical cohort	BAS<br>1	Initiation of treatment 86.3% control vs 94.5% intervention (P<0.05); delay 22.9 control days vs 5.6 intervention days; completion 57.4% control vs 68.1% intervention (P<0.01); interruption of treatment not significantly changed proportion of cured not significantly changed 8.5% control vs 9.9% intervention, (P=0.67); success rate (cure rate + completion rate) was significantly increased (OR: 0.54, Intervention 0.32-0.93, P=0.03).
J. A. Blaya	Peru	2010	P: Medical staff: N/A; Samples: Smear Microscopy Intervention 709, Control 561; Culture Intervention 697; Control 498<br>I: Notification system<br>C: Standard of care	CRCT<br>4	Intervention had 82% fewer errors than control for DST (2.1% vs. 11.9%, P < 0.001). For cultures, intervention had 87% fewer (2.0% vs. 15.1%, P < 0.001); missing paper results the same; users found results in the intervention that they did not have in control.
N. Mahmud	Malawi	2010	P: TB patients, intervention 200, control 100; Medical staff: 75<br>I: notification system<br>C: Standard of care, historical cohort	BAS<br>1	Net savings $2,750/6 months; total of 648 hours transport time for TB coordinator; 500 hours saved for nurse; doubled number of patients seen (100 -> 200); 2.11% technical problems.
L. Wang	China	2009	P: TB patients: 817 reported by hospital; 565 referred; 189 traced out of 229 needed tracing<br>I: TB patient tracing and notifications via phone calls<br>C: Standard of care, historical cohort	NRCT<br>2	Reported TB 42.5% control vs 95.3% intervention (P < 0.001); referred 48.1% control vs 83.3% intervention (P < 0.001); arrived at the medical facility 59.3% control vs 83.2% intervention (P < 0.001); arriving at the medical facility □3 days after reporting 38.4% control vs 66.2% intervention (P < 0.001); 71.7% increase in patients seen at the target hospitals. This is higher than the 25.8% increase in patients initially seen outside the target hospitals (P <0.001).
M. Zimic	Peru	2008	P: Microbiology samples: Mycobacterium tuberculosis: 50, Atypical Mycobacteria 20, Culture negative: 5<br>I: tele-microscopy with mobile phone<br>C: Standard of care, same cohort	BAS<br>2	98.7% concordance 74/75 (1 atypical mycobacteria). The single discrepancy corresponded to an atypical mycobacterium that was misclassified as a contaminant after mobile phone transmission.
J. A. Blaya	Peru	2008	P: Microscopy samples: Culture Intervention 1871, Control: 1679; Smear Microscopy intervention 2081, Control 1686	RCT<br>4	Time spent collecting & processing: 54% (5.45 to 2.52 min) for smear microscopy and by 66% (4.72 to 1.62 min) for cultures; Collection time: microscopy (1.36–2.11 min) and cultures (1.04–1.15 min) more vs control; Processing: intervention required 90% less time for smear microscopy (4.09–0.41 min) and 87%

			I: Notification system<br>C: Standard of care		less for cultures (3.68–0.47 min) vs control. Monetary break even point is 5.5 yrs. Satisfaction: mean 5/5 intervention vs mean 3.5/5 control. 1.13 /month technical problems staff could fix themselves.
B. Dwolatzky	South Africa	2006	P: TB patients, 20<br>I: Patient tracing & notifications using PDA/GPS<br>C: Standard of care, same cohort	BAS 1	Less crowded community 9/10 located homes on photograph; crowded community 6/10 located homes; both places with GPS 10/10 located homes.
S. B. Uldal	Russia	2005	P: TB patients: 47; Medical staff: 5 experts<br>I: Tele-consult<br>C: Standard of care, same cohort	BAS 1	Consensus >90%; 7 no difference in treatment; 22 wrong treatment avoided; 8 (17%) intervention revealed incorrect completion of local TB statistics; 1 wk-1mo saved for 24/47 (51%) cases; 1 case <1wk; 20 cases no change; faster in 37 cases; 30 (64%) saved trip to main city. In 10 cases (21%), the panel considered that the personnel increased their knowledge about a disease. 9 (19%), the panel considered that the requesting physician appeared to take more interest in computers than in the medical diagnosis.
S. B. Bavdekar	India	2005	P: Prospective TB Patients 200 (out of which 6 TB)<br>I: Internet-based diagnosis aid<br>C: Standard of care, same cohort	BAS 1	Tuberculosis: 6 cases, 4 matched by the diagnosis aid.
S. S. Choi	Peru	2004	P: Medical staff 7; TB patients: Intervention 95 (before) +102 (after), Control 92 (before) +81 (after)<br>I: notification system, reminders<br>C: Standard of care	NRCT 1	Medical record errors rates: error/patients intervention 17.4% December to 3.1% April (P=0.0075) vs control 8.6% December to 6.9% April (P=0.66). The nurses enjoyed working with the new system because it simplified workflow.
J. DeMaio	USA	2001	P: TB patients, 6<br>I: Video-DOT synchronous<br>C: Standard of care, same cohort	BAS 1	Adherence 97.5% on control & 95% on intervention. Up to 98% without technical problems. 16 doses of intervention missed: 6 not at home, 9 network (28k modem speed). Average satisfaction scores, 8.8 intervention and 8.4 control. Overall satisfaction intervention 9.2. Savings in travel expenses of $2870 and personnel expenses of $7993. A total of 8830 miles of travel saved. Quick (2–5 min), flexibility in scheduling.
P. Corr	South Africa	2000	P: intervention 27 prospective TB X-Rays (1 TB diagnosis), control 100<br>I: Tele-radiology via email<br>C: Standard of care, same cohort	BAS 1	96 diagnostic quality (96%) in controlC vs 23 (85%) in intervention; good correlation of diagnosis remote - centre 90 cases (94%) for control and 23 out of 27 images and a normal appearance in the other four images for intervention.
G. Hripcsak	USA	1999	P: Medical staff: 8; TB patients: 43<br>I: Diagnosis aid<br>C: Standard of care, same cohort	BAS 1	Positive pred value 0.96 (0.89-0.99); sensitivity of 0.89 (95% CI 0.75–0.96); Benefits: less paperwork, knowledge; nurse empowerment. Connection dropped from time to time. Some nurses complained the device was heavy. Patients trusted the privacy of the intervention.
P. Corr	South Africa	1997	P: TB patients 100; X-Rays: 75<br>I: Tele-radiology app and phone<br>C: Standard of care, same cohort	BAS 1	Altered management: detection of pulmonary tuberculosis (10 patients (5%)) and miliary tuberculosis (2 patients). Undiagnosed spinal tuberculosis was detected in 3 patients. Allowed same day reporting. Issues encountered with transmission and phone network.

Supplementary Table S2 PRISMA Checklist

Section and Topic	Item #	Checklist item	Location where item is reported
**TITLE**			
Title	1	Identify the report as a systematic review.	2
**ABSTRACT**			
Abstract	2	See the PRISMA 2020 for Abstracts checklist.	2
**INTRODUCTION**			
Rationale	3	Describe the rationale for the review in the context of existing knowledge.	3
Objectives	4	Provide an explicit statement of the objective(s) or question(s) the review addresses.	3
**METHODS**			
Eligibility criteria	5	Specify the inclusion and exclusion criteria for the review and how studies were grouped for the syntheses.	4
Information sources	6	Specify all databases, registers, websites, organisations, reference lists and other sources searched or consulted to identify studies. Specify the date when each source was last searched or consulted.	4
Search strategy	7	Present the full search strategies for all databases, registers and websites, including any filters and limits used.	S1
Selection process	8	Specify the methods used to decide whether a study met the inclusion criteria of the review, including how many reviewers screened each record and each report retrieved, whether they worked independently, and if applicable, details of automation tools used in the process.	4
Data collection process	9	Specify the methods used to collect data from reports, including how many reviewers collected data from each report, whether they worked independently, any processes for obtaining or confirming data from study investigators, and if applicable, details of automation tools used in the process.	4
Data items	10a	List and define all outcomes for which data were sought. Specify whether all results that were compatible with each outcome domain in each study were sought (e.g. for all measures, time points, analyses), and if not, the methods used to decide which results to collect.	5
	10b	List and define all other variables for which data were sought (e.g. participant and intervention characteristics, funding sources). Describe any assumptions made about any missing or unclear information.	5, S1
Study risk of bias assessment	11	Specify the methods used to assess risk of bias in the included studies, including details of the tool(s) used, how many reviewers assessed each study and whether they worked independently, and if applicable, details of automation tools used in the process.	5
Effect measures	12	Specify for each outcome the effect measure(s) (e.g. risk ratio, mean difference) used in the synthesis or presentation of results.	5
Synthesis methods	13a	Describe the processes used to decide which studies were eligible for each synthesis (e.g. tabulating the study intervention characteristics and comparing against the planned groups for each synthesis (item #5)).	5
	13b	Describe any methods required to prepare the data for presentation or synthesis, such as handling of missing summary statistics, or data conversions.	5

	13c	Describe any methods used to tabulate or visually display results of individual studies and syntheses.	5
	13d	Describe any methods used to synthesize results and provide a rationale for the choice(s). If meta-analysis was performed, describe the model(s), method(s) to identify the presence and extent of statistical heterogeneity, and software package(s) used.	5
	13e	Describe any methods used to explore possible causes of heterogeneity among study results (e.g. subgroup analysis, meta-regression).	5
	13f	Describe any sensitivity analyses conducted to assess robustness of the synthesized results.	5
Reporting bias assessment	14	Describe any methods used to assess risk of bias due to missing results in a synthesis (arising from reporting biases).	5
Certainty assessment	15	Describe any methods used to assess certainty (or confidence) in the body of evidence for an outcome.	5
**RESULTS**			
Study selection	16a	Describe the results of the search and selection process, from the number of records identified in the search to the number of studies included in the review, ideally using a flow diagram.	5
	16b	Cite studies that might appear to meet the inclusion criteria, but which were excluded, and explain why they were excluded.	5
Study characteristics	17	Cite each included study and present its characteristics.	S6-S16
Risk of bias in studies	18	Present assessments of risk of bias for each included study.	5, S5
Results of individual studies	19	For all outcomes, present, for each study: (a) summary statistics for each group (where appropriate) and (b) an effect estimate and its precision (e.g. confidence/credible interval), ideally using structured tables or plots.	6-8
Results of syntheses	20a	For each synthesis, briefly summarise the characteristics and risk of bias among contributing studies.	6-8
	20b	Present results of all statistical syntheses conducted. If meta-analysis was done, present for each the summary estimate and its precision (e.g. confidence/credible interval) and measures of statistical heterogeneity. If comparing groups, describe the direction of the effect.	6-8
	20c	Present results of all investigations of possible causes of heterogeneity among study results.	6-8
	20d	Present results of all sensitivity analyses conducted to assess the robustness of the synthesized results.	6-8
Reporting biases	21	Present assessments of risk of bias due to missing results (arising from reporting biases) for each synthesis assessed.	6-8
Certainty of evidence	22	Present assessments of certainty (or confidence) in the body of evidence for each outcome assessed.	12-14
**DISCUSSION**			
Discussion	23a	Provide a general interpretation of the results in the context of other evidence.	9
	23b	Discuss any limitations of the evidence included in the review.	9
	23c	Discuss any limitations of the review processes used.	9
	23d	Discuss implications of the results for practice, policy, and future research.	9
**OTHER INFORMATION**			

Registration and protocol	24a	Provide registration information for the review, including register name and registration number, or state that the review was not registered.	4
	24b	Indicate where the review protocol can be accessed, or state that a protocol was not prepared.	4
	24c	Describe and explain any amendments to information provided at registration or in the protocol.	-
Support	25	Describe sources of financial or non-financial support for the review, and the role of the funders or sponsors in the review.	10
Competing interests	26	Declare any competing interests of review authors.	10
Availability of data, code and other materials	27	Report which of the following are publicly available and where they can be found: template data collection forms; data extracted from included studies; data used for all analyses; analytic code; any other materials used in the review.	S1-19

From: Page MJ, McKenzie JE, Bossuyt PM, Boutron I, Hoffmann TC, Mulrow CD, et al. The PRISMA 2020 statement: an updated guideline for reporting systematic reviews. BMJ 2021;372:n71. doi: 10.1136/bmj.n71

For more information, visit: http://www.prisma-statement.org/
